# ICP22/IE63 Mediated Transcriptional Regulation and Immune Evasion: Two Important Survival Strategies for Alphaherpesviruses

**DOI:** 10.3389/fimmu.2021.743466

**Published:** 2021-12-02

**Authors:** Qing He, Ying Wu, Mingshu Wang, Shun Chen, Renyong Jia, Qiao Yang, Dekang Zhu, Mafeng Liu, Xinxin Zhao, Shaqiu Zhang, Juan Huang, Xumin Ou, Sai Mao, Qun Gao, Di Sun, Bin Tian, Anchun Cheng

**Affiliations:** ^1^ Institute of Preventive Veterinary Medicine, Sichuan Agricultural University, Chengdu, China; ^2^ Avian Disease Research Center, College of Veterinary Medicine of Sichuan Agricultural University, Chengdu, China; ^3^ Key Laboratory of Animal Disease and Human Health of Sichuan Province, Sichuan Agricultural University, Chengdu, China

**Keywords:** alphaherpesviruses, ICP22/IE63, RNA polymerase Pol II, immune evasion, antiviral response

## Abstract

In the process of infecting the host, alphaherpesviruses have derived a series of adaptation and survival strategies, such as latent infection, autophagy and immune evasion, to survive in the host environment. Infected cell protein 22 (ICP22) or its homologue immediate early protein 63 (IE63) is a posttranslationally modified multifunctional viral regulatory protein encoded by all alphaherpesviruses. In addition to playing an important role in the efficient use of host cell RNA polymerase II, it also plays an important role in the defense process of the virus overcoming the host immune system. These two effects of ICP22/IE63 are important survival strategies for alphaherpesviruses. In this review, we summarize the complex mechanism by which the ICP22 protein regulates the transcription of alphaherpesviruses and their host genes and the mechanism by which ICP22/IE63 participates in immune escape. Reviewing these mechanisms will also help us understand the pathogenesis of alphaherpesvirus infections and provide new strategies to combat these viral infections.

## 1 Introduction

Herpesviruses are a type of linear double-stranded DNA virus with the same morphology and capsule. At present, more than 120 kinds of herpesviruses have been identified that can infect humans and other vertebrates (1), and they mainly damage the skin, mucous membrane and nerve tissue and seriously affect the health of people and other animals. According to the different physical and chemical properties and biological characteristics of the viruses, herpesviruses can be divided into *Alphaherpesvirinae, Betaherpesvirinae, and Gammaherpesvirinae* (2). The human alphaherpesvirus (α-HV) subfamily includes herpes simplex virus type 1/2 (HSV-1/2) and varicella zoster virus (VZV). The animal α-HV subfamily includes pseudorabies virus (PRV), bovine herpesvirus (BHV), equine herpesvirus (EHV), Marek’s disease virus (GaHV-2), canine herpesvirus (CHV), and duck plague virus (DPV).

Previously, research on ICP22 mainly focused on its structure and transcriptional regulation function. Studies have shown that ICP22 is a transcription inhibitor that can restrict the transcription and expression of host genes that are not conducive to virus replication through different mechanisms during the transcription process (3). In recent years, it has been found that it also plays an important role in evading the host immune response. The important role played by ICP22/IE63 in transcriptional regulation and defense against host immunity has promoted the survival of α-HV. In this article, we describe the recent role of the ICP22/IE63 protein in viral and host gene transcription and its role in evading the host immune response. These effects are important strategies for the survival of α-HV *in vivo*. We aim to provide new treatment ideas for α-HV infection and the diseases it causes through an in-depth understanding of the molecular mechanism of ICP22/IE63 as a viral protein that hijacks host cell RNA polymerase II (Pol II) and resists the host’s immune response, finds and destroys the key targets of its molecular mechanism. Any drug that interferes with these steps may decrease viral survival, which will help develop new antiviral drugs and vaccines.

## 2 ICP22 Is Encoded by All Alphaherpesviruses

**Figure 1:**
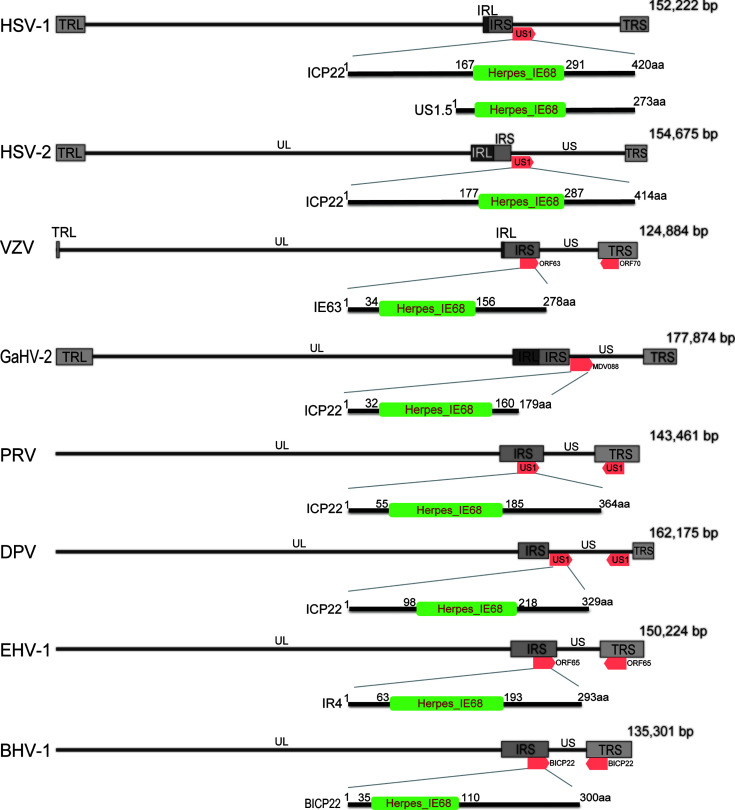
Schematic diagram of the position of ICP22 homologs in the α-HV genome. The α-HV genome is composed of unique long (UL), unique short (US), terminal repeat sequence (TRS) and internal repeat sequence (IRS). ICP22 homologs are located in the US or repeat sequence of the genome. The HSV-1, HSV-2, and GaHV-2 ICP22 genes are located in the US region of the viral genome and are single-copy genes; VZV, PRV, BHV-1, and EHV -1 ICP22 contain two copies, located in two inverted repeats of the genome. DPV ICP22 contains two copies, located in the US region of the genome. They all contain a conserved Herpes_IE68 domain, which is an immediate early protein.

At present, ICP22 research mainly focuses on HSV-1, HSV-2 and VZV. The ICP22 gene is found in a different location in the α-HV genome (**Figure 1**). According to reports, ICP22 is modified during viral infection, and its size is larger than the predicted molecular weight (4–9). For example, DPV ICP22 has a predicted molecular weight of 35 kDa but produces a widely modified 57 kDa protein in infected cells (7). In addition, VZV IE63 (ICP22 homolog) is encoded by open reading frames 63 and 70 (ORF63/70), with a predicted molecular weight of 30.5 kDa, but it produces a widely modified 45 kDa protein in infected cells (6). The genes of α-HV are divided into immediate early (IE) genes, early (E) genes and late (L) genes according to the timing of gene expression. IE genes can adjust the expression of E and L genes (10). ICP22 has been described as an IE gene in HSV-1, HSV-2, VZV and DPV but appears to be a nonIE gene in PRV (11) and both an IE and L gene in BoHV-1 and EHV-1 (8, 9, 12).

Bioinformatics analysis of the ICP22 protein showed that the α-HV ICP22 protein has a conserved Herpes_IE68 superfamily domain (**Figure 1**). Comparing the primary structure of the ICP22 protein encoded by α-HV, except for the conserved regions, the other regions have low homology (13) (**Table 1**). Studies have shown that aa 193-256 of HSV-1 ICP22 in the conserved sequence is sufficient to interact with cyclin-dependent kinase (CDK9) and inhibit Pol II carboxyl-terminal domain (CTD) serine (Ser)2 phosphorylation, which suggests that ICP22 from other α-HVs may also interact with and inhibit positive-transcription elongation factor b (P-TEFb), thereby mediating the transcriptional regulation of viral and host genes (3). The HSV-1 Us1 gene locus hides a second gene called Us1.5, which encodes a 273 amino acid N-terminal truncation of ICP22 (14). Us1.5 protein may have some regulatory effects on ICP22. Few studies have distinguished the function of ICP22 from the Us1.5 protein when analyzing ICP22 (15).

**Table 1:** Comparison of protein sequence similarity of ICP22 homologous protein of α-HV.

Homology Matrix of 8 sequences.								
HSV-1	100%							
HSV-2	73.1%	100%						
VZV	25.0%	26.3%	100%					
GaHV-2	20.5%	16.7%	25.6%	100%				
PRV	28.2%	26.9%	35.9%	26.3%	100%			
DEV	24.4%	22.4%	32.1%	26.9%	38.5%	100%		
EHV-1	22.4%	23.7%	38.5%	25.0%	57.1%	35.3%	100%	
BHV-1	21.8%	22.4%	34.0%	25.0%	50.0%	34.0%	53.2%	100%

In addition, the C-terminus of ICP22 has a large number of phosphorylation sites. Phosphorylation modification is essential for ICP22 to perform transcriptional regulation. After HSV-1 ICP22 is phosphorylated by the viral UL13 protein kinase, it participates in changing the phosphorylation status of Pol II, thereby promoting the transcriptional expression of late genes (16); the transcriptional inhibition region of VZV IE63 is located in the carboxy terminal region (210–278 aa), where CDK1-mediated phosphorylation of VZV IE63 Ser-224 and Thr-222 is essential to inhibit the basic activity of viral gene promoters, indicating that phosphorylation of IE63 is necessary for its suppressive properties (17–19).

Most ICP22 homologs have one or more nuclear localization signals (NLSs) (7, 9, 20–23), thus ICP22 is localized in the nucleus. Related studies have shown that after NLS mutation or deletion of HSV-1 ICP22 and DPV ICP22 (ΔICP22 NLS), or after infection with the ΔICP22 NLS virus, the ICP22 protein is still located in the nucleus (7, 22, 24), suggesting that ICP22 enters the nucleus. There may be a nonNLS-dependent mechanism, and it may be that ICP22 cooperates with some viral proteins or host proteins to localize it to the nucleus. GaHV-2 ICP22 does not contain an NLS, and it is located in the cytoplasm (25). Interestingly, the ectopic expression of GaHV-2 ICP22 downregulated the transcriptional activity of the five promoters tested (25), which indicates that GaHV-2 ICP22 may exert its inhibitory effect in the cytoplasm, that is, posttranscriptional level regulation. It has also been reported that after NLS mutation of VZV IE63, the transcriptional repressive function of IE63 is affected, but it still exerts a transcriptional repressive effect (18, 19). It is speculated that IE63 can exert its transcriptional inhibitory activity in two independent manners, namely, through transcription in the nucleus or a posttranscriptional mechanism in the cytoplasm.

## 3 ICP22 Protein Promotes Virus Survival Through Transcriptional Regulation

Viruses lack the basic mechanism of replication and must hijack the relevant functions of the host cell to complete the virus replication cycle, thereby producing progeny virus particles that can survive in the host. Similar to most nuclear-replicating DNA viruses, α-HV uses cellular Pol II to transcribe viral genes, thereby reproducing in host cells (26). ICP22 inhibits the Pol II occupancy rate of certain cell genes by participating in various mechanisms in transcriptional regulation and promotes the high-level transcription of viral genes by Pol II. Thereby promoting the survival of α-HV (27–29).

### 3.1 Eukaryotic Transcription Process Mediated by Pol II

**Figure 2:**
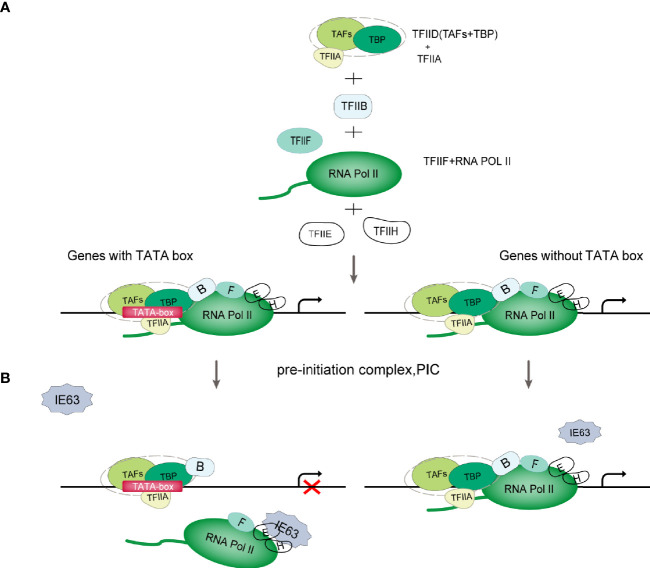
Schematic diagram of VZV IE63 hindering PIC assembly. **(A)** PIC assembly. The TFIID subunit TBP recognizes and binds to the TATA-box element on the promoter; then TFIIB binds to TBP, and TFIIB can also bind to DNA; then the TFIIB-TBP complex binds to the Pol II-TFIIF complex, and TFIIF can pass through Pol II interacts with TFIIB to reduce the binding of Pol II to the nonspecific part of DNA, thereby assisting Pol II to target binding to the promoter; finally, with the participation of TFIIE and TFIIH, PIC assembly is completed (31). **(B)** Schematic diagram of VZV IE63 affecting the assembly of the PIC. For genes containing a TATA box on the promoter, the ectopic expression of IE63 can interact with TFIIH, TFIIE and Pol II in the PIC on the promoter, creating steric obstacles to the assembly of the PIC, thereby interfering with the stability of the transcription initiation complex and inhibiting the initiation of transcription. In contrast, IE63 has no effect on genes without a TATA box.

The Pol II-mediated eukaryotic transcription process includes pretranscription initiation, transcription initiation, transcription extension and transcription termination (30). The initiation of gene transcription depends on the recognition of specific sequences in the promoter region and the assembly of the preinitiation complex (PIC) formed by cellular Pol II and a variety of transcription factors. The PIC is composed of Pol II and general transcription factors (GTFs) (**Figure 2A**). According to the presence or absence of the TATA box (TATA-box) on the gene promoters, genes can be divided into TATA-box-containing promoters or TATA-box-free promoters (32). The TATAA sequence is recognized by TATA-binding protein (TBP), which binds to DNA along with several TBP-associated factors (TAFs) as the TFIID complex. TFIIB recognizes BRE elements and facilitates the recruitment of hypophosphorylated Pol II-TFIIF and other GTFs to the promoter to assemble the PIC (**Figure 2A**). For genes lacking the typical TATA sequence, other core promoter elements play a major role in the recognition of the promoter by the transcription mechanism. TFIID and similar complexes can recognize such sequences, bind to DNA, and effectively form functional PICs (30) (**Figure 2A**). After the assembly of PIC is completed, the mediator complex activates TFIIH, leading to the phosphorylation of Ser-5 and Ser-7 of multiple repeated hepteptide sequences (Tyr1-Ser2-Pro3-Thr4-Ser5-Pro6-Ser7) on the C-terminal domain (CTD) of the large subunit of Pol II. Then, Pol II is released on the promoter to initiate transcription (33).

**Figure 3:**
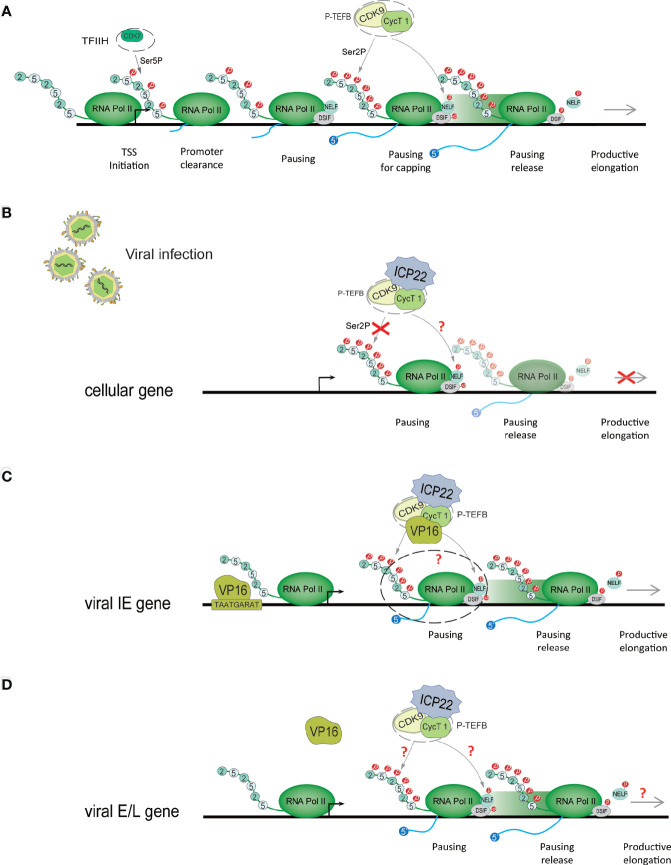
HSV-1 ICP22 changes the phosphorylation state of Pol II to regulate transcriptional extension. **(A)** Schematic diagram of the Pol II-mediated transcription process. When PIC is assembled, it stimulates the CDK7 subunit of TFIIH to phosphorylate CTD Ser-5. Immediately, Pol II breaks away from the gene promoter, and after the first 30-50 nucleotides are transcribed, it pauses at approximately 30-50 nucleotides downstream of the transcription start site (TSS). To overcome ppp, activated P-TEFB is recruited to the gene locus. Activated P-TEFb phosphorylates Pol II CTD Ser-2, NELF and DSIF and initiates Pol II’s efficient transcription extension mode. **(B)** HSV-1 ICP22 directly interacts with CDK9 and inhibits CDK9 enzyme activity, thereby inhibiting Ser-2 phosphorylation and inhibiting the transcription of cellular genes. **(C)** ICP22 regulates the IE gene. VP16 recognizes and binds to the core motif TAATGARAT near the IE gene promoter to activate IE gene expression. VP16 may release the transcriptional inhibition of the IE gene by ICP22 by interacting with P-TEFb. **(D)** It is unclear how HSV-1 blocks the inhibitory effect of ICP22 on E and L genes.

After Pol II starts transcription, it pauses approximately 30-50 nucleotides downstream of the transcription start site (TSS) (34, 35) (**Figure 3A**). This promoter-proximal pausing (PPP) serves as a checkpoint for gene transcription extension, ensuring the completion of the capping procedure at the 5’ end (36, 37). 1-β-D-ribofuranosylbenzimidazole sensitive inducing factor (DSIF) and negative elongation factor (NELF) are the key factors of PPP (38). NELFs and DSIFs can combine with Pol II in the transcription pause state and maintain the stability of their pause. To release Pol II and activate productive elongation, activated P-TEFb needs to be recruited into the gene locus (39). P-TEFb is composed of CDK9 and cyclin T1 (cycT1). Activated P-TEFb phosphorylates Pol II CTD Ser-2, NELF and DSIF, dissociates NELF from the transcription elongation complex, and reverses DSIF to a transcription elongation factor, thereby releasing Pol II in a state of transcriptional pause and initiating Pol II’s effective transcription extension mode (40–42). In this process, P-TEFb phosphorylation of Pol II may require the participation of other regulatory factors (40). Pol II transcription extends to the polyadenylation signal (PAS), and the synthesis ability of Pol II slows down. Various polyadenylation factors are recruited here to complete transcript cleavage and 3’ end processing (43).

### 3.2 VZV IE63 Inhibits the Expression of Certain Genes by Interfering With the Assembly of PIC to Increase the Production of Progeny Viruses

The role of IE63 as a transcriptional regulator is not fully understood and is still controversial. At present, most studies claim that IE63 is a transcription inhibitor, but some studies have shown that IE63 can activate the transcription of certain genes. The current consensus is that IE63 has limited transcriptional effects on viral and cellular genes (6, 25, 44–48). This effect depends in part on the cell type under study, viral vector and chromatin, and promoter accessibility (1, 25, 48). In addition, these inhibitory properties also depend on the phosphorylation state of the protein (18, 19, 48). Di Valentin E et al. (49) used VZV promoters to study the transcriptional regulation characteristics and mechanisms of the IE63 gene. Their results showed that IE63 can inhibit the basic activity of most viral promoters in epithelial cells and neuronal cells to varying degrees and can also inhibit the activity of heterologous viral genes and cellular gene promoters. The IE63-mediated inhibition mechanism is not controlled by the upstream regulatory elements of the promoter but only targets the TATA-box sequence. The basic activity of nonTATA-box promoters is not affected by IE63. In-depth research found that IE63 can interact with TFIIH, TFIIE and Pol II in PIC, resulting in a decrease in the content of TFIIB, TFIIE and TFIIH in PIC in the PIC assembly test. It is speculated that IE63 can block the interaction of PIC with many basic transcription factors and destroy the stability of PIC in this way. In addition, Habran et al. (48) assessed the regulatory characteristics of IE63 on the expression of endogenous genes based on the oligonucleotide-based microarray method and found that IE63 can positively or negatively regulate the transcription of some genes in HeLa cells, including genes related to transcription or immunity. This effect is mediated by modifying the binding of Pol II on the tested promoters. Therefore, in combination with the above research, we propose a model (**Figure 2B**): IE63 interacts with TFIIH, TFIIE and Pol II in PIC, creating steric obstacles to the assembly of PIC, interfering with the stability of the transcription initiation complex, and thereby inhibiting transcription initiation. This indicates that VZV IE63 is a transcription repressor that directly affects basic transcription factors. The assay also showed that IE63 does not affect TBP, and TBP recognition of TATA-box components is a key step in the formation of PIC. IE63 only interacts with a small number of GTFs, which also indicates that the transcriptional regulatory function of IE63 is limited. The mechanism of how IE63 targets the TATA-box sequence needs further study.

IE63 may regulate the expression of certain viral genes to promote viral survival. For example, IE63 clearly inhibits the transcription of the IE62 gene (25, 49). IE62 is the main transactivator of VZV and an essential gene for virus replication. IE62 has a negative transcriptional regulation effect on itself (50–52). It is speculated that maintaining IE62 below the cytotoxicity threshold is related to obtaining a high level of virus production and viral survival (53). Therefore, IE63 may promote viral survival by inhibiting the expression of the IE62 gene.

### 3.3 HSV-1 ICP22 Promotes Viral Survival by Regulating Transcription Elongation

#### 3.3.1 ICP22 Turns Off the Expression of Host Cell Genes That Are Not Conducive to Virus Replication by Regulating the CDK9-Mediated Pol II Phosphorylation Event in the P-TEFb Complex

HSV-1 significantly alters the phosphorylation modification of Pol II after infecting host cells, and ICP22 is a key protein involved in this effect (29, 54, 55). Pol II is composed of 12 subunits, and the amino acid sequence of the CTD of its LS consists of multiple repeating heptapeptide Tyr1-Ser2-Pro3-Thr4-Ser5-Pro6-Ser7 sequences. The CTD acts as a scaffold during transcription to recruit factors required for mRNA processing and chromatin modification of the Pol II complex, but this role depends on the phosphorylation of CTD (56), where the phosphorylation of CTD Ser-2 and Ser-5 are the most critical (**Figure 3A**). Phosphorylation of Ser-5 triggers recruitment of the capping enzyme for formation of the 5´ cap structure. Ser-2 phosphorylation is closely related to Pol II’s ability to overcome transtranscriptional pauses and can serve as a key point for transcriptional regulation (57). In addition to its role in transcription elongation, Ser-2 phosphorylation also recruits cleavage and polyadenylation factors for the generation of the mRNA 3´ end (58, 59), and it has been implicated in recruiting splicing and mRNA export factors (60, 61).

Pol II CTD Ser2 phosphorylation is mediated by P-TEFb. The CDK9 subunit of P-TEFb provides enzymatic activity, while cyclin has a regulatory role (62, 63). Many elongation regulators play a role in regulating transcription elongation by interacting with P-TEFb (64). Multiple studies have shown that HSV-1 ICP22 directly interacts with CDK9 and inhibits CDK9 enzyme activity, thereby inhibiting Ser-2 phosphorylation and inhibiting the transcription of cellular genes (3, 29, 65) (**Figure 3B**).

ICP22 mediates the loss of Ser-2 phosphorylation on the Pol II CTD, which not only inhibits host cell gene transcription but also affects the expression of viral genes (66). The HSV-1 virus can overcome this effect through other mechanisms. Studies have shown that ectopic expression of ICP22 can inhibit the transcription of HSV-1 IE (ICP4), E (TK), and L (gC, VHS) promoter reporter genes. Studies have shown that VP16 activates the transcription of IE genes by recognizing the core motif TAATGARAT in the promoter sequence of IE genes and binding to the promoter of IE genes (67, 68). The coexpression of VP16 and ICP22 protein indicates that VP16 can eliminate the transcriptional inhibition of ICP22 on the IE genes (65, 69). Immunoprecipitation assays showed that VP16 can interact with P-TEFb, but there was no direct interaction with ICP22 (29). ICP22, P-TEFb and VP16 form the ICP22-P-TEFb-VP16 multiprotein complex in the cell. This suggests that the cooperative regulation of viral genes by ICP22 and VP16 is related to P-TEFb. We speculate that VP16 interacts with P-TEFb to relieve the transcriptional inhibitory effect of ICP22 on IE genes (**Figure 3C**). However, the specific mechanism of cooperative regulation of VP16 and ICP22 is still unclear. The latest research shows that VP16 competes with ICP22 to interact with P-TEFB, thereby eliminating the inhibition of P-TEFb by ICP22 (29). In addition, studies have shown that ICP22 is necessary for the efficient transcription of viral L genes in some cell lines (54), but it is unclear how HSV-1 eliminates the inhibitory effects of ICP22 on E and L genes (**Figure 3D**). It is possible that after VP16 releases the transcriptional suppression of the IE gene by ICP22, the activated IE proteins ICP0, ICP4 and ICP27 are beneficial to the transcriptional expression of the E and L genes, but this requires further research and verification. However, it has been suggested that HSV-1 transcription may not require the Ser-2 phosphorylated form of Pol II CTD, although this form is necessary for cellular transcriptional extension and RNA processing (70). However, experiments have verified that HSV-1 transcription requires Ser-2 phosphorylation of the Pol II CTD (70). In addition, inhibition of Pol II CTD Ser2 phosphorylation suggests that NELF and DSIF phosphorylation may also be inhibited, but this remains to be determined. CDK9 also phosphorylates p53, a tumor suppressor that plays a central role in the response of cells to a range of stress factors. The interaction of ICP22 with the CDK9 target p53 is also important for efficient HSV-1 replication (71).

Several studies have provided maps of Pol II localization on the virus and host genome after HSV-1 infection (27, 28). HSV-1 infection leads to extensive transcription termination defects in host cell genes (72). Studies have shown that Pol II was almost completely removed from two-thirds of the host genes in the early stage of HSV-1 infection. The function of a few genes that increase the occupancy rate of Pol II is related to the upregulation of exosomal secretion and the downregulation of apoptosis, which may be beneficial to virus production. The HSV-1 genome contains a large amount of Pol II, which reflects the high-level transcription of viral genes. However, the Pol II occupancy level in the genome of the ICP22 mutant virus is reduced, and the accumulation of mRNA for almost all viral genes is reduced (73). Among them, ICP22 may downregulate cellular gene expression and promote viral gene expression by regulating the CDK9-mediated Pol II phosphorylation event in the P-TEFb complex, thereby promoting viral survival. ICP22 is not the only viral factor that affects Pol II during HSV-1 infection, which is the synergistic effect of HSV-1 gene products (74). This highlights the transcriptional regulation of the HSV-1 protein to maximize the expression of the viral genome while downregulating the expression of the host genome. As a result, the expression of host cell genes that are not conducive to virus replication is turned off, and the antiviral immune response of the host is destroyed. Therefore, this promotes the production of viral infectious virus particles.

#### 3.3.2 HSV-1 ICP22 Promotes the Production of Infectious Virus Particles by Recruiting FACT Complexes

**Figure 4:**
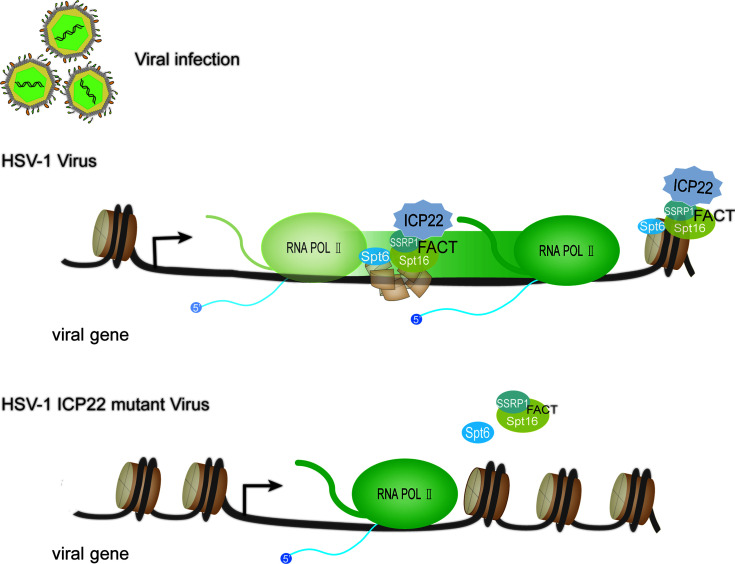
HSV-1 ICP22 affects the efficiency of transcriptional extension through FACT recruitment. In the presence of ICP22, ICP22 can recruit the FACT complex to the viral genome by interacting with the FACT complex, thereby promoting Pol II to cross the nucleosome barrier on the viral genome and achieve effective transcription extension. When the HSV-1 ICP22 mutant infects cells, the number of FACT complex subunits and Spt6 in the genome of the ICP22 mutant is significantly reduced.

Nucleosomes uniquely positioned on high-affinity DNA sequences present a polar barrier to transcription by Pol II (75). When the Pol II transcription elongation complex extends to the gene body, it needs to cross the nucleosome barrier (76, 77). There are a variety of regulatory mechanisms in cells that can remove or weaken the nucleosome barrier to assist Pol II extension, such as nucleosome remodeling and histone modification. Currently, a number of regulatory factors involved in regulating Pol II transcription elongation complex crossing nucleosome barriers have been identified *in vitro* using biochemical experiments, such as Facilitates chromatin transcription (FACT), Spt6, PAF complex and PARP (76). FACT was originally isolated and identified from an extract of the human HeLa cell nucleus (78), and it is a complex composed of SSRP1 and Spt16. FACT functions to disassemble an H2A-H2B dimer from nucleosomes, and Pol II can be transcribed through the remaining histone hexamer without being displaced (62, 79). When the Pol II transcription complex passes through the nucleosome, FACT can also promote the reassembly of the H2A-H2B dimer and the remaining hexamer into the histone octamer and maintain a highly activated state (80). Studies have found that after HSV-1 infects host cells, FACT relocates to the virus replication compartment in the nucleus, and FACT is abundant in the HSV-1 genome during the replication of HSV-1 (81, 82). This change is related to ICP22 protein (29, 73). Studies have shown that FACT interacts with ICP22 throughout infection. Transcriptome sequencing (RNA-seq) and chromatin immunoprecipitation-sequencing (ChIP-seq) experiments showed that compared with wild-type, regardless of the type of kinetics, the accumulation of almost all viral mRNAs of ICP22 mutant virus late infection was reduced. The Pol II occupancy level on the mutant virus genome is reduced (73). In contrast, the association of Pol II with the transcription initiation site in the mutant was not decreased. This shows that in the absence of ICP22, the viral gene transcription elongation rate is reduced. This indicates that ICP22 can recruit elongation factors (such as the FACT complex) into the HSV-1 genome to play a role and achieve effective viral transcription elongation. This promotes the production of infectious virus particles in late viral infection. Except for the FACT complex, in the absence of ICP22, the number of transcription elongation factors (Spt6 and Spt5) recruited by the viral genome was significantly reduced. These proteins all promote the elongation of cellular transcription and are known to interact with Pol II (83, 84). Based on the above research, we developed a model (**Figure 4**): when HSV-1 infects cells, ICP22 can recruit FACT complexes to the viral genome by interacting with the FACT complex, thereby promoting Pol II to cross the nucleosome barrier on the viral genome and achieve efficient viral transcription elongation late in viral infection and ultimately infectious virion production.

## 4 ICP22 Protein Promotes Viral Survival Through an Anti-Host Antiviral Response

### 4.1 HSV-2 ICP22 Inhibits Key Steps of the Host Type I IFN (Interferon) Pathway

**Figure 5:**
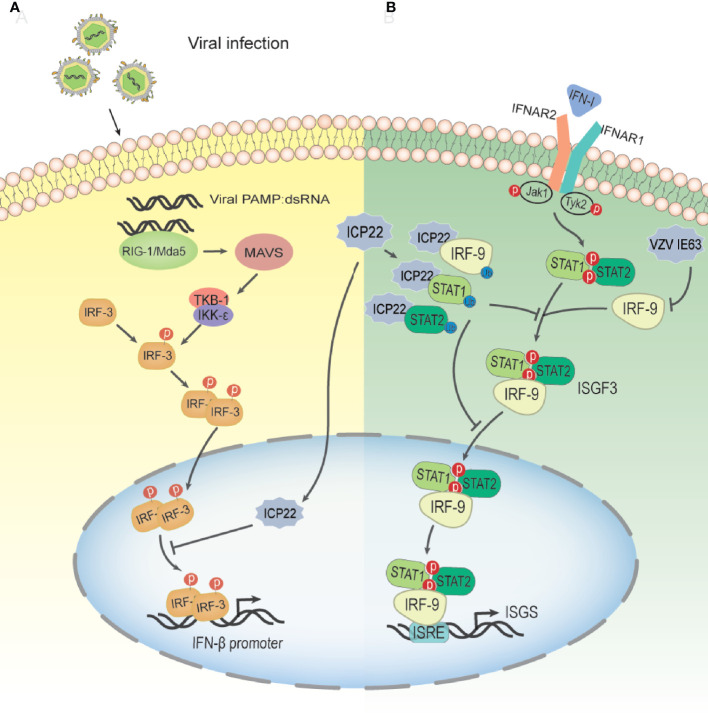
Schematic diagram of HSV-2 ICP22 inhibiting the host type I IFN pathway. **(A)** The mechanism by which HSV-2 blocks the IRF-3 signal transduction pathway. HSV-2 infection yields a number of byproducts, such as dsRNA, which can be recognized by RIG-I and activate the RIG-I/Mda-5 pathway, thereby promoting IFN-β transcription. Under HSV-2 infection conditions, ICP22 interacts with the DNA binding domain of IRF-3, resulting in the suppression of IRF-3 association with the IFN-β promoter. Ultimately, the production of IFN-β is suppressed by HSV-2, contributing to viral immune evasion(94). **(B)** The mechanism by which HSV-2 ICP22 blocks the IFN-β-mediated signaling pathway. Type I IFNs are usually expressed at low levels and can be induced by viral infection. After IFN-1 binds to receptors on the cell surface, Jak1 and Tyk2 are activated, leading to tyrosine phosphorylation of STAT1 and STAT2. Phosphorylated STATs dimerize and associate with IRF9 to form ISGF3. ISGF3 translocates to the nucleus and binds to ISRE to activate the transcription of ISG so that the cell is in an antiviral state. After HSV-2 infection, ICP22 induces ubiquitination and degradation of STAT1, STAT2 and IRF9, thereby blocking ISGF3 nuclear translocation. Finally, the production of ISG is inhibited by HSV-2, which promotes the immune escape of HSV-2(100). VZV IE63 interferes with type I IFN-mediated activation of JAK-STAT signaling by degrading IRF9, thereby inhibiting the expression of interferon-stimulated genes.

Type I IFNs are vital cytokines in controlling viral infections (85). The induction of the type I IFN response during virus infection includes two stages. The first stage is the production of type I interferon (86–88); the second stage is the induction of IFN-stimulated genes (ISGs) (89, 90). After the viral infection trigger signal, retinoic acid inducible gene I (RIG-I)/melanoma-associated differentiation gene 5 (MDA-5) binds to dsRNA, recruits and activates the expression of mitochondrial antiviral signaling (MAVS) protein (91, 92). Then, activated MAVS can activate IKK-ϵ/TBK-1, which in turn leads to phosphorylation and dimerization of IRF-3, resulting in IRF-3 dimers translocating from the cytoplasm to the nucleus. Subsequently, activated IRF-3 and other coactivators combine with the positive regulatory region upstream of the IFN-β promoter to enhance IFN-β transcription in a synergistic mode (93) (**Figure 5A**). IRF-3 is a key transcription factor in the type I IFN production pathway. IRF-3 consists of 427 amino acids, including a DNA binding domain (aa 1–112) responsible for DNA binding and an IRF association domain (IAD; aa 197–394) responsible for IRF-3 phosphorylation, dimerization, and interaction with the CBP/p300 coactivator (94). Studies have shown that in the case of HSV-2 infection, ICP22 interacts with the DNA binding domain of IRF-3, resulting in the suppression of the binding of IRF-3 to the IFN-β promoter and a decrease in the expression of IFN-β(6A) (95). The 217-414 domain of ICP22 is sufficient to inhibit the production of IFN-β at a level similar to that of full-length ICP22. Only a few viral components have been reported to interfere with the binding of activated IRF-3 to the IFN-β promoter, including human Boca virus nuclear protein NP1 and Kaposi sarcoma-associated herpesvirus latency-associated nuclear antigen Ag (94, 96). Surprisingly, HSV-1 ICP22, which shares approximately 70% of the amino acid sequence with HSV-2 ICP22, has no such inhibitory effect on IFN-β production. However, some HSV-1 proteins can interfere with IRF-3-mediated signaling pathways through other mechanisms (97–100). HSV-2 ICP22 inhibits the production of IFN-β by blocking the binding of IRF-3 to the IFN-β promoter, contributing to viral immune evasion. This may be one of the strategies by which HSV-2 evades the host’s innate immune response.

In addition to inhibiting the production of IFN-β, the latest research shows that HSV-2 ICP22, as a novel E3, induces the ubiquitination of multiple proteins, resulting in the blockade of type I IFN signaling (101). As shown in **Figure 5B**, HSV-2 ICP22 acts as an E3 ubiquitin protein ligase to interact with STAT1, STAT2, IRF9 and other ubiquitinated proteins and induces ubiquitination and degradation of STAT1, STAT2 and IRF9, thereby blocking ISG factor 3 (ISGF3) nuclear translocation. Ultimately, the production of ISGs is suppressed by HSV-2 (101). These findings emphasize a new mechanism through which HSV-2 circumvents the host antiviral response through the viral E3 ubiquitin protein ligase. Interestingly, although a previous study showed that HSV-1 ICP22 did not have the same effect on IFN-β induction as HSV-2 ICP22 (95), this study showed that HSV-1 ICP22 could suppress the activation of ISG54 and ISG56 transcription.

### 4.2 HSV-1 ICP22 Participates in the Mechanism of Virus Immune Evasion

#### 4.2.1 HSV-1 ICP22 Weakens the Host’s Immune Response in Dendritic Cells by Downregulating CD80 Expression

**Figure 6:**
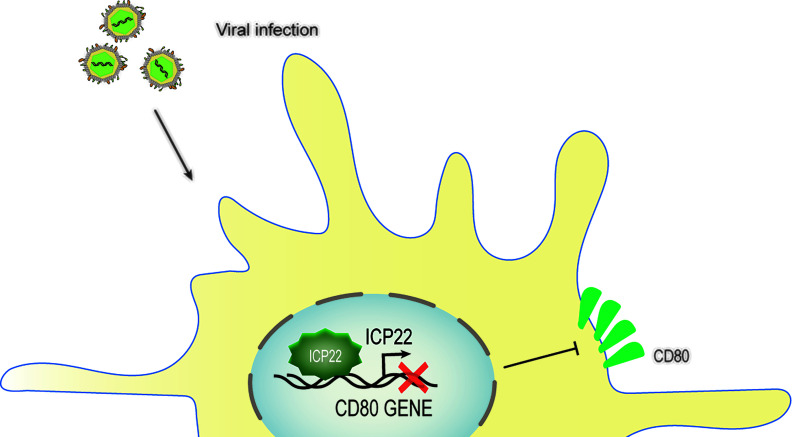
Schematic diagram of HSV-1 ICP22 downregulating CD80 expression. Ocular infection of mice with HSV-1 suppressed the expression of the costimulatory molecule CD80. HSV-1 ICP22 downregulates the expression of CD80 by specifically binding to the CD80 promoter.

HSV-1 ocular infections are the most common cause of corneal blindness in developed countries (102). Recurrent infections and the prolonged inflammatory response after viral clearance both contribute to corneal scarring (103). HSV-1 infection is recognized by antigen-presenting cells such as dendritic cells (DCs), natural killer cells, and macrophages (104, 105), which induce the secretion of IFNs and cytokines and the activation of CD4+ and CD8+ T cells. This T cell activation is strictly controlled and requires at least two signals, which involve the binding of CD28, CTLA-4 or PD-1 on the surface of T cells corresponding to the APC costimulatory molecule CD80 (B7-1) or CD86 (B7-2) (106). The costimulatory molecule CD80/86 drives T-cell activation and proliferation by binding to CD28 (107). CD80 plays a critical role in increased inflammatory responses in HSV-1-infected mouse corneas (108). Studies have shown that increasing CD80 levels promote increased CD8+ T cells, leading to exacerbated eye disease in HSV-1-infected mice (109). A study described a new mechanism of HSV-1 immune escape through ICP22-dependent downregulation of the host T cell costimulatory molecule CD80 in DCs (110, 111). According to reports, ocular infection of mice with HSV-1 suppressed the expression of the costimulatory molecule CD80 but not CD86 in the cornea (110). This effect was specifically mediated by the binding of HSV-1 ICP22 to the CD80 promoter, which is located between positions 151 and 462 of the CD80 promoter, and this interaction was required for HSV-1-mediated inhibition of CD80 expression. The latest research shows that the binding site of ICP22 and the CD80 promoter is located at aa 305–345 of ICP22. The use of HSV-1 recombinant virus expressing truncated ICP22 lacking CD80 promoter binding increased the expression of CD80 in DCs and the expression of IFN-γ in CD8+ T cells but did not increase CD4+ T cells in mouse corneas (111). Other viral proteins, such as ICP0, ICP27 or ICP47, can significantly increase CD80 promoter activity, and these increases did not offset the inhibition of CD80 by ICP22. The large increase in CD80 promoter activity observed in ICP22-deficient HSV-1 virus-infected DCs indicates that only ICP22 can counteract the stimulatory effect of other IE genes on CD80 expression, and no other HSV-1 genes have the effect of inhibiting CD80 expression. In contrast, overexpression of CD80 by infecting mouse eyes with recombinant HSV-1 lacking ICP22 will exacerbate corneal scarring in the infected mice, leading to greater eye disease. Interestingly, studies have shown that although D22 (recombinant virus lacking ICP22) replicates poorly in the eyes of infected mice, the level of T-cell infiltration caused by D22 infection is similar to that of its parental strain WT KOS (108). HSV-1 uses the ICP22-CD80 promoter interaction to specifically downregulate CD80 as a key immune escape mechanism and weakens the host’s immune response (109) (**Figure 6**). ICP22 plays a critical role in reducing HSV-1-mediated immunopathology.

#### 4.2.2 HSV-1 ICP22, as a Virus-Encoded Chaperone Protein (J-Protein/Hsp40), May Be Involved in Chaperone-Mediated Autophagy

It was previously reported that HSV-1 ICP22 prevents the aggregation of nonnative proteins and recruits cell heat shock protein 70 (Hsc70) into the nuclear domain (112, 113). Recent studies have shown that ICP22 resembles a cellular J-protein/HSP40 family cochaperone, interacting specifically with Hsc70 (114). These findings showed that ICP22 reduced cytoplasmic protein aggregation, and ICP22 provided protection against the heat inactivation of firefly luciferase. Sequence homology analysis indicated that ICP22 contains an N-terminal J-domain and a C-terminal substrate binding domain, similar to type II cellular J-proteins. ICP22 may thus be functionally similar to J-protein/Hsp40 cochaperones that function together with their HSP70 partners to prevent aggregation of nonnative proteins. HSP40/HSP70 complexes have been shown to play critical roles in a myriad of cellular processes, including the regulation of gene expression and cell cycle control (115–117). This also suggests that ICP22 may be involved in chaperone-mediated autophagy (CAM) as a cochaperone protein similar to HSP40 and Hsc70. The CMA process involves three stages: recognition of molecular chaperones and substrates, unfolding of substrates and translocation across lysosomal membranes (118). This process requires the participation of two protein complexes: one is the substrate recognition complex, and the other is the lysosomal transmembrane translocation complex (118). The substrate recognition complex is composed of Hsc70 and other cochaperone molecules, including HSP90, HSP40, Hip, Hop and Bag-1, and recognizes protein substrate molecules with KFERQ sequences (118–120). Among them, Hsp40 stimulates the ATPase activity of Hsc70, leading to increased rates of binding and release of substrate proteins (118). In addition, Hsc70 is also involved in other antiviral defense mechanisms, such as aggregate formation and proteolytic degradation of viral proteins (121, 122). During HSV-1 infection, the ability of ICP22 to relocate Hsc70 to the VICE domain in the nucleus may prevent Hsc70 from exerting antiviral activity in other parts of the cell (113). Therefore, we speculate that ICP22, as a common partner similar to the cell J protein/HSP40 family, may participate in some functions of Hsc70 in this manner, but this needs to be verified by further experiments. This is the first known example of a virus obtaining a complete J-like protein, indicating that HSV has used the adaptability of the J protein to evolve a multifunctional helper chaperone that works with Hsc70 to promote lytic infection.

#### 4.2.3 The Effect of HSV-1 ICP22 on Cell Apoptosis

It is generally believed that ICP22 not only promotes apoptosis but can also inhibit apoptosis. HSV-1 in the absence of ICP22 induced more apoptotic cells, indicating that ICP22 can block apoptosis (123, 124). However, the antiapoptotic activity of ICP22 was not strong. Therefore, ICP22-mediated antiapoptosis is unlikely to directly act on apoptosis signal transduction but precisely regulate the expression of the E and L genes (including antiapoptotic genes) of the virus. For example, in the absence of ICP22 in HSV-1, the expression of US5, which inhibits apoptosis, is delayed (125–127).

In addition, ICP22 was cleaved by caspases in cells infected with HSV-1 d120 (apoptosis inducer), revealing another product called Mr 37500 (128). This process can be inhibited by overexpression of Bcl-2, transfection with US3 or addition of caspase-3 inhibitors. In the process of viral infection, the cleavage of viral proteins by caspases can lead to a variety of consequences, such as the adverse effect of apoptosis, the enhancement or reduction of replication, and the spread of the virus (129, 130).

In addition, HSV-1 ICP22 can interact with p53 and antagonize p53 (71). p53 is a key cellular transcription factor that plays a central role in cellular responses to a broad range of stress factors through its regulation of a variety of cellular pathways, such as apoptosis, the cell cycle, cellular senescence, DNA repair, autophagy, and innate immune control (131, 132). Studies have shown that p53 plays a positive role in HSV-1 replication. However, p53 has no effect on the replication of the ICP22-deficient strain (71). It is speculated that the effect of p53 on HSV-1 replication depends on ICP22, but this requires experimental verification. In addition, p53 promotes the expression of ICP27 in early infection without relying on ICP22, but the negative effect of p53 on reducing the expression of ICPO in late infection can be offset by ICP22. However, it is not clear whether ICP22 can directly regulate cell apoptosis by interacting with p53.

On the other hand, the antiapoptotic effects of HSV-1 US1.5 and ICP22 during infection were the opposite. Overexpression of US1.5 with a baculovirus vector has been shown to trigger the activation of caspase 3 in rabbit skin cells (133). This result indicates that US1.5 may have proapoptotic activity (130).

### 4.3 VZV IE63 Participates in the Mechanism of Virus Immune Evasion

#### 4.3.1 VZV IE63 Modulates Proinflammatory Gene Transcription by Inhibiting the NF-KB Pathway

**Figure 7:**
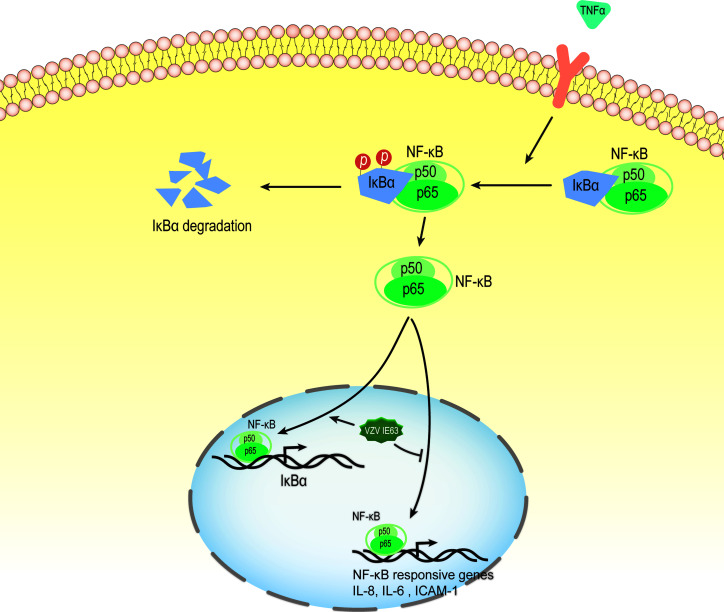
VZV IE63 modulates proinflammatory gene transcription by inhibiting the NF-KB pathway. The activation of NF-κB signaling is caused by extracellular stimulation. These stimuli are recognized by the receptor and delivered to the cell, which ultimately leads to the activation of IκB kinase (IKK). IKK phosphorylates inhibitory IκB in the cytoplasm and this causes IκB to be degraded by the proteasome and releases NF-κB from the inhibitory complex. The released NF-κB protein is then transported to the nucleus, where it binds to the target sequence DNA and activates gene transcription. After treating HeLa cells with TNFα, the presence of IE63 reduced the expression of IL-8, IL-6 and ICAM-1 mediated by TNFα and increased the expression of the IκBα gene. IE63 expressed in TNFα-treated HeLa cells reduced the binding of p65 to the NF-κB proximal sites on the IL-8 and ICAM-1 promoters and enhanced the recruitment of p65 to the IκBα promoter. Treatment of IE63-expressing cells with TNFα inhibits the NF-κB pathway, suggesting that VZV may be a strategy to resist VZV-infected cells from exogenous proinflammatory cytokine-induced antiviral reactions.

The nuclear factor kappa-B (NF-κB) transcription factor family can be found in almost all animal cells, and they are involved in the response of cells to external stimuli. NF-κB transcription factors are activated in response to a variety of signals, including cytokines, pathogens, injury, and other stress conditions. The activation of this factor leads to the expression of several immune response genes, such as proinflammatory cytokines (IFN-β, TNF-α, IL-6, IL-8), chemokines and adhesion molecules. Therefore, NF-κB plays a key role in the inflammatory response and immune response of cells (134, 135). In unstimulated cells, NF-κB binds to the inhibitory protein IκB and isolates the NF-κB·IκB complex in the cytoplasm, thereby preventing NF-κB from binding to DNA. The activation of NF-κB signaling is caused by extracellular stimulation. These stimuli are recognized by the receptor and transmitted to the cell, which ultimately leads to the activation of IκB kinase (IKK). IKK phosphorylates the inhibitory IκB subunit of the NF-κB·IκB complex in the cytoplasm. This phosphorylation causes IκB to be degraded by the proteasome and releases NF-κB from the inhibitory complex (136, 137). The released NF-κB protein is then transported to the nucleus, where it binds to the target sequence DNA and activates gene transcription (**Figure 7**).

Studies have reported that transiently expressed VZV IE63 can downregulate the activity of several cellular NF-κB-responsive gene promoters, such as IL-8 and IL-6 (49). Subsequently, Habran et al. (48) showed that IE63 does not change the basic expression of these genes, which may be due to the issue of promoter accessibility related to chromatin. After treating HeLa cells with TNFα, a cytokine known to increase the utilization of several chromatins, the researchers measured the transcription levels of these genes (IL-8, IL-6, ICAM-1 and IκBα). The results showed that the presence of IE63 can reduce the expression of IL-8, IL-6 and ICAM-1 mediated by TNFα and increase the expression of the IκBα gene. In addition, after the degradation of IκBα, IE63 can promote the resynthesis of IκBα. This result indicates that the presence of IE63 promotes complementation of the IκBα library. The phosphorylation status of IE63 is crucial for affecting TNFα-mediated gene transcription. When alanine replaces the phosphorylation site, IE63-S224/T222A does not significantly regulate TNFα-induced gene transcription. Chip assays showed that IE63 reduced the accessibility of IL-8 and ICAM-1 promoters and increased the accessibility of TNFα to IκBα promoters. IE63 expressed in TNFα-treated HeLa cells reduced the binding of p65 to the NF-κB proximal sites on the IL-8 and ICAM-1 promoters and enhanced the recruitment of p65 to the IκBα promoter. It is speculated that the inhibition of IL-8 and ICAM-1 gene expression may be the result of IκBα induction because IκBα is an inhibitor of NF-κB (136). IE63 interferes with the TNF-inducing ability of several NF-κB-dependent genes through the accelerated resynthesis of IκBα. Although the molecular mechanism of these effects has not yet been determined, the treatment of IE63-expressing cells with TNFα inhibits the NF-κB pathway, suggesting that VZV may use such a strategy to resist VZV-infected cells from exogenous proinflammatory cytokine-induced antiviral reactions.

#### 4.3.2 VZV IE63 Interferes With Type I IFN Signaling by Inhibiting the JAK-STAT Signaling Pathway and eIF-2α Phosphorylation

**Figure 8:**
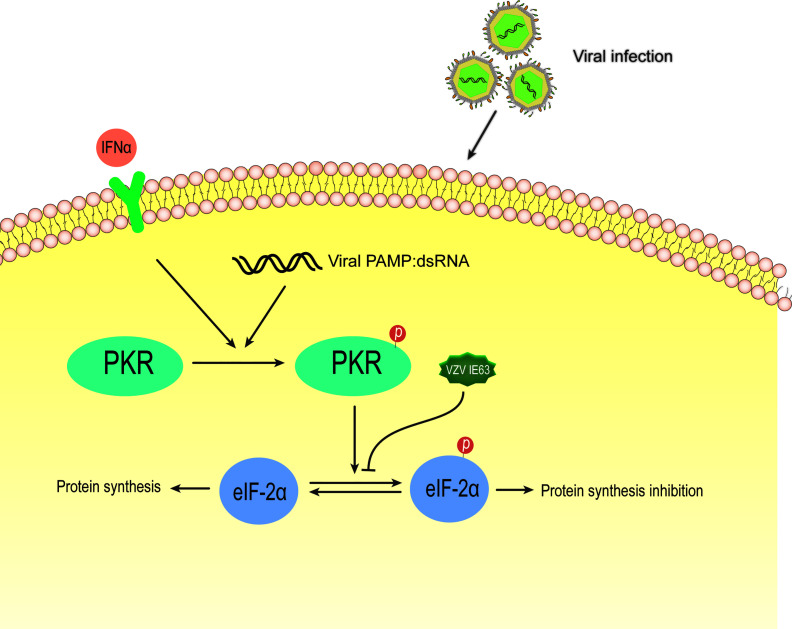
Schematic diagram of VZV IE63 regulating protein synthesis in infected cells. During most viral infections, double-stranded RNA is synthesized or a double-stranded RNA structure is formed. After IFN-induced PKR kinase is activated by double-stranded RNA (dsRNA), activated PKR phosphorylates eIF-2α, and its phosphorylation causes translational inhibition, thereby inhibiting virus replication. The phosphorylation level of eIF-2α increases in cells infected with the IE63 mutant. The eIF-2α phosphorylation level of cells transiently expressing IE63 decreases, indicating that IE63 can promote the expression of VZV virus by inhibiting eIF-2α phosphorylation.

Ambagala and Cohen reported that VZV IE63 is required to inhibit IFN-α-induced antiviral responses (138), and they used IE63 deletion virus to infect human melanoma cells and U2OS cells. The results showed that the virus is highly sensitive to the antiviral effect of human IFN-α but not to IFN-γ compared to the parental and other viral gene mutants. IFN-α inhibited the expression of viral genes in cells infected with IE63 deletion virus at the posttranscriptional level without affecting its mRNA level. An important component of the innate response enhanced by the activity of IFNs is the signaling of the double-stranded RNA sensor PKR (139). PKR is an IFN-induced, double-stranded RNA (dsRNA)-activated serine/threonine protein kinase (140). This latent enzyme needs to be activated by autophosphorylation. Unless blocked, activated PKR phosphorylates the alpha subunit of eukaryotic initiation factor 2 (eIF-2α), whose phosphorylation causes inhibition of translation and, therefore, inhibition of virus replication (141). Most viruses either synthesize double-stranded RNA or form a double-stranded RNA structure during infection, thereby activating PKR. Viruses have evolved several mechanisms to interfere with eIF-2α phosphorylation and prevent the inhibition of protein synthesis in infected cells (142). In HSV-1, PKR is blocked by several viral genes, which redirect protein phosphatase 2 (PP2A) to dephosphorylate eIF-2α (143, 144). It has been reported that cells infected with the IE63 mutant have increased eIF-2 phosphorylation compared to cells infected by the parental virus (138). In the same study, cells transiently expressing IE63 showed a decrease in the basal level of eIF-2α phosphorylation, indicating that IE63 is sufficient to inhibit this phosphorylation (**Figure 8**). These results indicate that IE63 may inhibit eIF-2α phosphorylation through the PKR sensor pathway, resist IFN-α-induced inhibition of protein synthesis and promote viral protein synthesis and virus replication.

The deletion of IE63 makes the deletion virus sensitive to IFN-α(the replication of the deletion virus is significantly reduced), suggesting that IE63 may be involved in the regulation of JAK-STAT signaling (138). The latest research shows that VZV inhibits the signal transduction activated by type I IFN through the JAK-STAT pathway. VZV infection leads to a decrease in IRF9 and a decrease in STAT2 protein and its phosphorylation (145). IE63 is the key protein for this change. In the presence of IFN, the expression of IE63 alone led to a reduction in IRF9 protein, while STAT2 did not change. The expression of IE63 led to a decrease in the expression of luciferase induced by IFNs. This result indicated that VZV IE63 interferes with type I IFN-mediated activation of JAK-STAT signaling by reducing IRF9, thereby inhibiting the expression of interferon-stimulated genes (**Figure 5**). Simian varicella virus (SVV) shares approximately 75% DNA homology with VZV and exhibits a highly similar genome organization (146). SVV IE63, which is homologous to VZV IE63, inhibits the JAK-STAT pathway in a similar manner and degrades IRF9 in a proteasome-dependent manner. HSV-2 ICP22, as an E3 protease, participates in the proteasome to degrade IRF9 and STATs, thereby interfering with the signal transduction of type I IFN. Unlike HSV-2, VZV IE63 does not affect STAT2 and its phosphorylation, and there may be other proteins encoded by VZV that are responsible for inhibiting STAT2 phosphorylation and reducing STAT2 expression. Whether the degradation mechanism of VZV IE63 IRF9 is the same as that of HSV-2 ICP22 needs to be verified through experiments. It has not been reported that VZV IE63 has the effect of the E3 protease, and its specific mechanism needs to be further studied. The above studies show that VZV IE63 plays a key role in regulating the innate immune response to VZV.

#### 4.3.3 The Effect of VZV IE63 on Cell Apoptosis

VZV IE63 can also block apoptosis (147, 148). rOkaORF63 (a recombinant virus unable to express one copy of the diploid IE gene) (ORF63)-infected neurons were more susceptible to apoptosis than parental rOka-infected neurons. Furthermore, the expression of IE63 protein in neurons alone can resist apoptosis induced by nerve growth factor (NGF) (149). These results showed that IE63 can suppress apoptosis of neurons. Compared with parental virus, IE63-deleted VZV has higher levels of phosphorylated eIF-2α to promote apoptosis. Meanwhile, the expression of IE63 alone can be sufficient to block eIF-2α phosphorylation (130, 138). This indicates that during active VZV infection, IE63 participates in a protective mechanism against apoptosis in neurons. Although this study was limited to productive infections, the rich expression of IE63 during latent infection suggests that this gene product may play an anti-apoptotic function during the incubation period or reactivation of neurons (150, 151). Similar to HSV-1 ICP22, VZV IE63 can inhibit cell apoptosis, but it functions through a completely different mechanism. The specific mechanism by which VZV IE63 inhibits cell apoptosis still needs further study.

## 5 Summary

In alphaherpesviruses, ICP22 is a transcriptional regulatory protein that promotes viral survival by participating in multiple transcriptional regulatory processes: VZV IE63 can interact with certain PIC components including Pol II, TFIIH, and TFIIE to limit the transcriptional start of cellular genes; HSV-1 ICP22 can inhibit its subunits by interacting with P-TEFB CDK9, which phosphorylates Pol II, thereby inhibiting transcription pause release; HSV-1 ICP22 can promote viral gene transcription extension by recruiting FACT to the viral genome in the viral replication compartment. Alphaherpesviruses have corresponding strategies to release the inhibitory effect of ICP22 on viral genes. For example, HSV-1 VP16 can release the inhibitory effect of ICP22 on the IE gene through interaction with P-TEFb. The final manifestation is that in the early stage of viral infection, ICP22 downregulates the expression of host genes and promotes high-level transcription of viral genes. As a result, the expression of host cell genes that are not conducive to virus replication is turned off, and the host’s antiviral immune response is destroyed. These complex mechanisms involved in ICP22 provide a foundation and challenge for us to understand these mechanisms and develop various potential treatment options. In addition, ICP22 destroys the host’s antiviral immune response in a variety of ways and promotes viral survival. In the future, the relationship between ICP22 and viruses and hosts can be further analyzed by using ICP22 genetically engineered mutants combined with deep sequencing, such as PRO-seq. Current *in vitro* cell assays cannot fully reveal all the functions of ICP22. ICP22 mutants should continue to be used in animal models to further reveal the regulation and mechanism of ICP22 on host and viral gene transcription *in vivo*, as well as the antiviral effect of ICP22. With the deepening of the understanding of viral proteins, it will inject new vitality into the treatment of herpesviruses and the development of new vaccines.

## Author Contributions

QH and YW contributed to the design and writing of the article. MW, RJ, SC, QY, DZ, ML, XZ, SZ, JH, XO, SM, QG, DS, and BT all provided ideas contributing to the structure of this article. AC modified the article. All authors contributed to the article and approved the submitted version.

## Funding

This research was supported by the China Agriculture Research System of MOF and MARA and the Program Sichuan Veterinary Medicine and Drug Innovation Group of the China Agricultural Research System (SCCXTD-2021-18).

## Conflict of Interest

The authors declare that the research was conducted in the absence of any commercial or financial relationships that could be construed as a potential conflict of interest.

## Publisher’s Note

All claims expressed in this article are solely those of the authors and do not necessarily represent those of their affiliated organizations, or those of the publisher, the editors and the reviewers. Any product that may be evaluated in this article, or claim that may be made by its manufacturer, is not guaranteed or endorsed by the publisher.

## References

[1] McGeoch DJ Rixon FJ Davison AJ. Topics in Herpesvirus Genomics and Evolution. Virus Res (2006) 117(1):90–104. doi: 10.1016/j.virusres.2006.01.002 16490275

[2] Davison AJ Eberle R Ehlers B Hayward GS McGeoch DJ Minson AC. The Order Herpesvirales. Arch Virol (2009) 154(1):171–7. doi: 10.1007/s00705-008-0278-4 PMC3552636 19066710

[3] Zaborowska J Baumli S Laitem C O’Reilly D Thomas PH O’Hare P. Herpes Simplex Virus 1 (HSV-1) ICP22 Protein Directly Interacts With Cyclin-Dependent Kinase (CDK)9 to Inhibit RNA Polymerase II Transcription Elongation. PloS One (2014) 9(9):e107654. doi: 10.1371/journal.pone.0107654 25233083 PMC4169428

[4] Kolb AW Schmidt TR Dyer DW. Brandt CR. Sequence Variation in the Herpes Simplex Virus U(S)1 Ocular Virulence Determinant. Invest Ophthalmol Visual Sci (2011) 52(7):4630–8. doi: 10.1167/iovs.10-7032 PMC3175936 21519032

[5] Boumart I Figueroa T Dambrine G Muylkens B Pejakovic S Rasschaert D. GaHV-2 ICP22 Protein is Expressed From a Bicistronic Transcript Regulated by Three GaHV-2 microRNAs. J Gen Virol (2018) 99(9):1286–300. doi: 10.1099/jgv.0.001124 30067174

[6] Baiker A Bagowski C Ito H Sommer M Zerboni L Fabel K. The Immediate-Early 63 Protein of Varicella-Zoster Virus: Analysis of Functional Domains Required for Replication *In Vitro* and for T-Cell and Skin Tropism in the SCIDhu Model *In Vivo* . J Virol (2004) 78(3):1181–94. doi: 10.1128/jvi.78.3.1181-1194.2004 PMC321405 14722273

[7] Li Y Wu Y Wang M Ma Y Jia R Chen S. Duplicate US1 Genes of Duck Enteritis Virus Encode a Non-Essential Immediate Early Protein Localized to the Nucleus. Front Cell Infect Microbiol (2019) 9:463. doi: 10.3389/fcimb.2019.00463 32010642 PMC6979402

[8] Kim SK Holden VR O’Callaghan DJ. The ICP22 Protein of Equine Herpesvirus 1 Cooperates With the IE Protein to Regulate Viral Gene Expression. J Virol (1997) 71(2):1004–12. doi: 10.1128/JVI.71.2.1004-1012.1997 PMC191150 8995619

[9] Köppel R Vogt B Schwyzer M. Immediate-Early Protein BICP22 of Bovine Herpesvirus 1 Trans-Represses Viral Promoters of Different Kinetic Classes and is Itself Regulated by BICP0 at Transcriptional and Posttranscriptional Levels. Arch Virol (1997) 142(12):2447–64. doi: 10.1007/s007050050254 9672606

[10] Honess RW Roizman B. Regulation of Herpesvirus Macromolecular Synthesis. I. Cascade Regulation of the Synthesis of Three Groups of Viral Proteins. J Virol (1974) 14(1):8–19. doi: 10.1128/jvi.14.1.8-19.1974 4365321 PMC355471

[11] Tombacz D Toth JS Petrovszki P Boldogkoi Z. Whole-Genome Analysis of Pseudorabies Virus Gene Expression by Real-Time Quantitative RT-PCR Assay. BMC Genomics (2009) 10:491. doi: 10.1186/1471-2164-10-491 19852823 PMC2775753

[12] Slater JD Gibson JS Field HJ. Pathogenicity of a Thymidine Kinase-Deficient Mutant of Equine Herpesvirus 1 in Mice and Specific Pathogen-Free Foals. J Gen Virol (1993) 74(Pt 5):819–28. doi: 10.1099/0022-1317-74-5-819 8388018

[13] Li Y Wu Y Wang M Cheng A. Research Progress of Herpes Virus ICP22 Protein. Acta Veterinaria Et Zootechnica Sin (2021) 52(1):9–18. doi: 10.11843/j.issn.0366-6964.2021.01.002

[14] Bowman JJ Schaffer PA. Origin of Expression of the Herpes Simplex Virus Type 1 Protein U(S)1.5. J Virol (2009) 83(18):9183–94. doi: 10.1128/jvi.00984-09 PMC2738232 19570862

[15] Mostafa HH Davido DJ. Herpes Simplex Virus 1 ICP22 But Not US 1.5 Is Required for Efficient Acute Replication in Mice and VICE Domain Formation. J Virol (2013) 87(24):13510–9. doi: 10.1128/JVI.02424-13 PMC3838291 24089574

[16] Long MC Leong V Schaffer PA Spencer CA Rice SA. ICP22 and the UL13 Protein Kinase Are Both Required for Herpes Simplex Virus-Induced Modification of the Large Subunit of RNA Polymerase II. J Virol (1999) 73(7):5593–604. doi: 10.1128/jvi.73.7.5593-5604.1999 PMC112617 10364308

[17] Mueller NH Walters MS Marcus RA Graf LL Prenni J Gilden D. Identification of Phosphorylated Residues on Varicella-Zoster Virus Immediate-Early Protein ORF63. J Gen Virol (2010) 91(Pt 5):1133–7. doi: 10.1099/vir.0.019067-0 PMC2888152 20089801

[18] Bontems S Di Valentin E Baudoux L Rentier B Sadzot-Delvaux C Piette J. Phosphorylation of Varicella-Zoster Virus IE63 Protein by Casein Kinases Influences its Cellular Localization and Gene Regulation Activity. J Biol Chem (2002) 277(23):21050–60. doi: 10.1074/jbc.M111872200 11912195

[19] Habran L Bontems S Di Valentin E Sadzot-Delvaux C Piette J. Varicella-Zoster Virus IE63 Protein Phosphorylation by Roscovitine-Sensitive Cyclin-Dependent Kinases Modulates its Cellular Localization and Activity. J Biol Chem (2005) 280(32):29135–43. doi: 10.1074/jbc.M503312200 15955820

[20] Cai M Jiang S Zeng Z Li X Mo C Yang Y. Probing the Nuclear Import Signal and Nuclear Transport Molecular Determinants of PRV Icp22. Cell Biosci (2016) 6:3. doi: 10.1186/s13578-016-0069-7 26816613 PMC4727382

[21] Walters MS Kyratsous CA Wan S Silverstein S. Nuclear Import of the Varicella-Zoster Virus Latency-Associated Protein ORF63 in Primary Neurons Requires Expression of the Lytic Protein ORF61 and Occurs in a Proteasome-Dependent Manner. J Virol (2008) 82(17):8673–86. doi: 10.1128/JVI.00685-08 PMC2519623 18562514

[22] Stelz G Rücker E Rosorius O Meyer G Stauber RH Spatz M. Identification of Two Nuclear Import Signals in the Alpha-Gene Product ICP22 of Herpes Simplex Virus 1. Virology (2002) 295(2):360–70. doi: 10.1006/viro.2002.1384 12033795

[23] Holden VR Caughman GB Zhao Y Harty RN O’Callaghan DJ. Identification and Characterization of the ICP22 Protein of Equine Herpesvirus 1. J Virol (1994) 68(7):4329–40. doi: 10.1128/jvi.68.7.4329-4340.1994 PMC236356 8207808

[24] Bastian TW Rice SA. Identification of Sequences in Herpes Simplex Virus Type 1 ICP22 That Influence RNA Polymerase II Modification and Viral Late Gene Expression. J Virol (2009) 83(1):128–39. doi: 10.1128/jvi.01954-08 PMC2612302 18971282

[25] Hoover SE Cohrs RJ Rangel ZG Gilden DH Munson P Cohen JI. Downregulation of Varicella-Zoster Virus (VZV) Immediate-Early ORF62 Transcription by VZV ORF63 Correlates With Virus Replication *In Vitro* and With Latency. J Virol (2006) 80(7):3459–68. doi: 10.1128/jvi.80.7.3459-3468.2006 PMC1440367 16537613

[26] Sandri-Goldin RM. Initiation of Transcription and RNA Synthesis, Processing and Transport in HSV and VZV Infected Cells. In: Arvin A Campadelli-Fiume G Mocarski E Moore PS Roizman B Whitley R. editors. Human Herpesviruses: Biology, Therapy, and Immunoprophylaxis. Cambridge: Cambridge University Press (2007). 21348061

[27] Durand LO Advani SJ Poon AP Roizman B. The Carboxyl-Terminal Domain of RNA Polymerase II is Phosphorylated by a Complex Containing Cdk9 and Infected-Cell Protein 22 of Herpes Simplex Virus 1. J Virol (2005) 79(11):6757–62. doi: 10.1128/jvi.79.11.6757-6762.2005 PMC1112163 15890914

[28] Abrisch RG Eidem TM Yakovchuk P Kugel JF Goodrich JA. Infection by Herpes Simplex Virus 1 Causes Near-Complete Loss of RNA Polymerase II Occupancy on the Host Cell Genome. J Virol (2015) 90(5):2503–13. doi: 10.1128/JVI.02665-15 PMC4810688 26676778

[29] Isa NF Bensaude O Aziz NC Murphy S. HSV-1 ICP22 Is a Selective Viral Repressor of Cellular RNA Polymerase II-Mediated Transcription Elongation. Vaccines (2021) 9(10):1054. doi: 10.3390/vaccines9101054 PMC8539892 34696162

[30] Shandilya J Roberts SG. The Transcription Cycle in Eukaryotes: From Productive Initiation to RNA Polymerase II Recycling. Biochim Biophys Acta (2012) 1819(5):391–400. doi: 10.1016/j.bbagrm.2012.01.010 22306664

[31] Nikolov DB Burley SK. RNA Polymerase II Transcription Initiation: A Structural View. Proc Natl Acad Sci USA (1997) 94(1):15–22. doi: 10.1073/pnas.94.1.15 8990153 PMC33652

[32] Baumann M Pontiller J Ernst W. Structure and Basal Transcription Complex of RNA Polymerase II Core Promoters in the Mammalian Genome: An Overview. Mol Biotechnol (2010) 45(3):241–7. doi: 10.1007/s12033-010-9265-6 20300884

[33] Guo J Price DH. RNA Polymerase II Transcription Elongation Control. Chem Rev (2013) 113(11):8583–603. doi: 10.1021/cr400105n PMC4294624 23919563

[34] Danko CG Hah N Luo X Martins AL Core L Lis JT. Signaling Pathways Differentially Affect RNA Polymerase II Initiation, Pausing, and Elongation Rate in Cells. Mol Cell (2013) 50(2):212–22. doi: 10.1016/j.molcel.2013.02.015 PMC3640649 23523369

[35] Adelman K Lis JT. Promoter-Proximal Pausing of RNA Polymerase II: Emerging Roles in Metazoans. Nat Rev Genet (2012) 13(10):720–31. doi: 10.1038/nrg3293 PMC3552498 22986266

[36] Kwak H Fuda NJ Core LJ Lis JT. Precise Maps of RNA Polymerase Reveal How Promoters Direct Initiation and Pausing. Science (2013) 339(6122):950–3. doi: 10.1126/science.1229386 PMC3974810 23430654

[37] Mahat DB Kwak H Booth GT Jonkers IH Danko CG Patel RK. Base-Pair-Resolution Genome-Wide Mapping of Active RNA Polymerases Using Precision Nuclear Run-on (PRO-Seq). Nat Protoc (2016) 11(8):1455–76. doi: 10.1038/nprot.2016.086 PMC5502525 27442863

[38] Cucinotta CE Arndt KM. SnapShot: Transcription Elongation. Cell (2016) 166(4):1058–1058.e1051. doi: 10.1016/j.cell.2016.07.039 27518568

[39] Jonkers I Lis JT. Getting Up to Speed With Transcription Elongation by RNA Polymerase II. Nat Rev Mol Cell Biol (2015) 16(3):167–77. doi: 10.1038/nrm3953 PMC4782187 25693130

[40] Yu M Yang W Ni T Tang Z Nakadai T Zhu J. RNA Polymerase II-Associated Factor 1 Regulates the Release and Phosphorylation of Paused RNA Polymerase II. Science (2015) 350(6266):1383–6. doi: 10.1126/science.aad2338 PMC8729149 26659056

[41] Peterlin BM Price DH. Controlling the Elongation Phase of Transcription With P-TEFb. Mol Cell (2006) 23(3):297–305. doi: 10.1016/j.molcel.2006.06.014 16885020

[42] Ni Z Saunders A Fuda NJ Yao J Suarez JR Webb WW. P-TEFb is Critical for the Maturation of RNA Polymerase II Into Productive Elongation *In Vivo* . Mol Cell Biol (2008) 28(3):1161–70. doi: 10.1128/MCB.01859-07 PMC2223398 18070927

[43] Proudfoot NJ. Transcriptional Termination in Mammals: Stopping the RNA Polymerase II Juggernaut. Science (2016) 352(6291):aad9926. doi: 10.1126/science.aad9926 27284201 PMC5144996

[44] Jackers P Defechereux P Baudoux L Lambert C Massaer M Merville-Louis MP. Characterization of Regulatory Functions of the Varicella-Zoster Virus Gene 63-Encoded Protein. J Virol (1992) 66(6):3899–903. doi: 10.1128/jvi.66.6.3899-3903.1992 PMC241178 1316489

[45] Kost RG Kupinsky H Straus SE. Varicella-Zoster Virus Gene 63: Transcript Mapping and Regulatory Activity. Virology (1995) 209(1):218–24. doi: 10.1006/viro.1995.1246 7747473

[46] Kenyon TK Lynch J Hay J Ruyechan W Grose C. Varicella-Zoster Virus ORF47 Protein Serine Kinase: Characterization of a Cloned, Biologically Active Phosphotransferase and Two Viral Substrates, ORF62 and ORF63. J Virol (2001) 75(18):8854–8. doi: 10.1128/jvi.75.18.8854-8858.2001 PMC115131 11507231

[47] Zuranski T Nawar H Czechowski D Lynch JM Arvin A Hay J. Cell-Type-Dependent Activation of the Cellular EF-1alpha Promoter by the Varicella-Zoster Virus IE63 Protein. Virology (2005) 338(1):35–42. doi: 10.1016/j.virol.2005.05.005 15936796

[48] Habran L El Mjiyad N Di Valentin E Sadzot-Delvaux C Bontems S Piette J. The Varicella-Zoster Virus Immediate-Early 63 Protein Affects Chromatin-Controlled Gene Transcription in a Cell-Type Dependent Manner. BMC Mol Biol (2007) 8:99. doi: 10.1186/1471-2199-8-99 17971236 PMC2176069

[49] Di Valentin E Bontems S Habran L Jolois O Markine-Goriaynoff N Vanderplasschen A. Varicella-Zoster Virus IE63 Protein Represses the Basal Transcription Machinery by Disorganizing the Pre-Initiation Complex. Biol Chem (2005) 386(3):255–67. doi: 10.1515/bc.2005.031 15843171

[50] Perera LP Mosca JD Sadeghi-Zadeh M Ruyechan WT Hay J. The Varicella-Zoster Virus Immediate Early Protein, IE62, Can Positively Regulate Its Cognate Promoter. Virology (1992) 191(1):346–54. doi: 10.1016/0042-6822(92)90197-w 1329324

[51] Sato B Ito H Hinchliffe S Sommer MH Zerboni L Arvin AM. Mutational Analysis of Open Reading Frames 62 and 71, Encoding the Varicella-Zoster Virus Immediate-Early Transactivating Protein, IE62, and Effects on Replication *In Vitro* and in Skin Xenografts in the SCID-Hu Mouse *In Vivo* . J Virol (2003) 77(10):5607–20. doi: 10.1128/jvi.77.10.5607-5620.2003 PMC154054 12719553

[52] White K Peng H Hay J Ruyechan WT. Role of the IE62 Consensus Binding Site in Transactivation by the Varicella-Zoster Virus IE62 Protein. J Virol (2010) 84(8):3767–79. doi: 10.1128/jvi.02522-09 PMC2849489 20130051

[53] Chaturvedi S Engel R Weinberger L. The HSV-1 ICP4 Transcriptional Auto-Repression Circuit Functions as a Transcriptional “Accelerator” Circuit. Front Cell Infect Microbiol (2020) 10:265. doi: 10.3389/fcimb.2020.00265 32670890 PMC7326776

[54] Rice SA Long MC Lam V Schaffer PA Spencer CA. Herpes Simplex Virus Immediate-Early Protein ICP22 Is Required for Viral Modification of Host RNA Polymerase II and Establishment of the Normal Viral Transcription Program. J Virol (1995) 69(9):5550–9. doi: 10.1128/jvi.69.9.5550-5559.1995 PMC189408 7637000

[55] Rice SA Long MC Lam V Spencer CA. RNA Polymerase II Is Aberrantly Phosphorylated and Localized to Viral Replication Compartments Following Herpes Simplex Virus Infection. J Virol (1994) 68(2):988–1001. doi: 10.1128/JVI.68.2.988-1001.1994 8289400 PMC236537

[56] Heidemann M Hintermair C Voß K Eick D. Dynamic Phosphorylation Patterns of RNA Polymerase II CTD During Transcription. Biochim Biophys Acta (2013) 1829(1):55–62. doi: 10.1016/j.bbagrm.2012.08.013 22982363

[57] Li J Gilmour DS. Promoter Proximal Pausing and the Control of Gene Expression. Curr Opin Genet Dev (2011) 21(2):231–5. doi: 10.1016/j.gde.2011.01.010 PMC3443551 21324670

[58] Gu B Eick D Bensaude O. CTD Serine-2 Plays a Critical Role in Splicing and Termination Factor Recruitment to RNA Polymerase II *In Vivo* . Nucleic Acids Res (2013) 41(3):1591–603. doi: 10.1093/nar/gks1327 PMC3561981 23275552

[59] Davidson L Muniz L West S. 3’ End Formation of Pre-mRNA and Phosphorylation of Ser2 on the RNA Polymerase II CTD Are Reciprocally Coupled in Human Cells. Genes Dev (2014) 28(4):342–56. doi: 10.1101/gad.231274.113 PMC3937513 24478330

[60] Rice SA Davido DJ. HSV-1 ICP22: Hijacking Host Nuclear Functions to Enhance Viral Infection. Future Microbiol (2013) 8(3):311–21. doi: 10.2217/fmb.13.4 23464370

[61] Saldi T Cortazar MA Sheridan RM Bentley DL. Coupling of RNA Polymerase II Transcription Elongation With Pre-mRNA Splicing. J Mol Biol (2016) 428(12):2623–35. doi: 10.1016/j.jmb.2016.04.017 PMC4893998 27107644

[62] Bondarenko MT Maluchenko NV Valieva ME Gerasimova NS Kulaeva OI Georgiev PG. Structure and Function of Histone Chaperone FACT. Molekuliarnaia Biol (2015) 49(6):891–904. doi: 10.7868/s0026898415060026 26710768

[63] De Luca A De Falco M Baldi A Paggi MG. Cyclin T: Three Forms for Different Roles in Physiological and Pathological Functions. J Cell Physiol (2003) 194(2):101–7. doi: 10.1002/jcp.10196 12494448

[64] Zaborowska J Isa NF Murphy S. P-TEFb Goes Viral. Inside Cell (2016) 1(2):106–16. doi: 10.1002/icl3.1037 PMC4863834 27398404

[65] Guo L Wu WJ Liu LD Wang LC Zhang Y Wu LQ. Herpes Simplex Virus 1 ICP22 Inhibits the Transcription of Viral Gene Promoters by Binding to and Blocking the Recruitment of P-TEFb. PloS One (2012) 7(9):e45749. doi: 10.1371/journal.pone.0045749 23029222 PMC3454370

[66] Bowman JJ Orlando JS Davido DJ Kushnir AS Schaffer PA. Transient Expression of Herpes Simplex Virus Type 1 ICP22 Represses Viral Promoter Activity and Complements the Replication of an ICP22 Null Virus. J Virol (2009) 83(17):8733–43. doi: 10.1128/JVI.00810-09 PMC2738139 19535441

[67] LaBoissière S Walker S O’Hare P. Concerted Activity of Host Cell Factor Subregions in Promoting Stable VP16 Complex Assembly and Preventing Interference by the Acidic Activation Domain. Mol Cell Biol (1997) 17(12):7108–18. doi: 10.1128/mcb.17.12.7108 PMC232567 9372942

[68] Lai JS Herr W. Interdigitated Residues Within a Small Region of VP16 Interact With Oct-1, HCF, and DNA. Mol Cell Biol (1997) 17(7):3937–46. doi: 10.1128/mcb.17.7.3937 PMC232246 9199328

[69] Cun W Guo L Zhang Y Liu L Wang L Li J. Transcriptional Regulation of the Herpes Simplex Virus 1α-Gene by the Viral Immediate-Early Protein ICP22 in Association With VP16. Sci China Ser C: Life Sci (2009) 52(4):344–51. doi: 10.1007/s11427-009-0051-2 19381460

[70] Ou M Sandri-Goldin RM. Inhibition of Cdk9 During Herpes Simplex Virus 1 Infection Impedes Viral Transcription. PloS One (2013) 8(10):e79007. doi: 10.1371/journal.pone.0079007 24205359 PMC3799718

[71] Maruzuru Y Fujii H Oyama M Kozuka-Hata H Kato A Kawaguchi Y. Roles of P53 in Herpes Simplex Virus 1 Replication. J Virol (2013) 87(16):9323–32. doi: 10.1128/jvi.01581-13 PMC3754062 23785201

[72] Rutkowski AJ Erhard F L’Hernault A Bonfert T Schilhabel M Crump C. Widespread Disruption of Host Transcription Termination in HSV-1 Infection. Nat Commun (2015) 6:7126. doi: 10.1038/ncomms8126 25989971 PMC4441252

[73] Fox HL Dembowski JA DeLuca NA. A Herpesviral Immediate Early Protein Promotes Transcription Elongation of Viral Transcripts. mBio (2017) 8(3):e00745–17. doi: 10.1128/mBio.00745-17 PMC5472187 28611249

[74] Dremel SE DeLuca NA. Herpes Simplex Viral Nucleoprotein Creates a Competitive Transcriptional Environment Facilitating Robust Viral Transcription and Host Shut Off. Elife (2019) 8:e51109. doi: 10.7554/eLife.51109 PMC6805162 31638576

[75] Bondarenko VA Steele LM Ujvári A Gaykalova DA Kulaeva OI Polikanov YS. Nucleosomes can Form a Polar Barrier to Transcript Elongation by RNA Polymerase II. Mol Cell (2006) 24(3):469–79. doi: 10.1016/j.molcel.2006.09.009 17081995

[76] Kwak H Lis JT. Control of Transcriptional Elongation. Annu Rev Genet (2013) 47(1):483–508. doi: 10.1146/annurev-genet-110711-155440 24050178 PMC3974797

[77] Kobayashi W Kurumizaka H. Structural Transition of the Nucleosome During Chromatin Remodeling and Transcription. Curr Opin Struct Biol (2019) 59:107–14. doi: 10.1016/j.sbi.2019.07.011 31473439

[78] LeRoy G Orphanides G Lane WS Reinberg D. Requirement of RSF and FACT for Transcription of Chromatin Templates *In Vitro* . Science (1998) 282(5395):1900–4. doi: 10.1126/science.282.5395.1900 9836642

[79] Belotserkovskaya R Oh S Bondarenko VA Orphanides G Studitsky VM Reinberg D. FACT Facilitates Transcription-Dependent Nucleosome Alteration. Science (2003) 301(5636):1090–3. doi: 10.1126/science.1085703 12934006

[80] Xin H Takahata S Blanksma M McCullough L Stillman DJ Formosa T. yFACT Induces Global Accessibility of Nucleosomal DNA Without H2A-H2B Displacement. Mol Cell (2009) 35(3):365–76. doi: 10.1016/j.molcel.2009.06.024 PMC2748400 19683499

[81] Dembowski JA DeLuca NA. Selective Recruitment of Nuclear Factors to Productively Replicating Herpes Simplex Virus Genomes. PloS Pathog (2015) 11(5):e1004939. doi: 10.1371/journal.ppat.1004939 26018390 PMC4446364

[82] Dembowski JA Dremel SE DeLuca NA. Replication-Coupled Recruitment of Viral and Cellular Factors to Herpes Simplex Virus Type 1 Replication Forks for the Maintenance and Expression of Viral Genomes. PloS Pathog (2017) 13(1):e1006166. doi: 10.1371/journal.ppat.1006166 28095497 PMC5271410

[83] Swanson MS Winston F. SPT4, SPT5 and SPT6 Interactions: Effects on Transcription and Viability in Saccharomyces Cerevisiae. Genetics (1992) 132(2):325–36. doi: 10.1093/genetics/132.2.325 PMC1205139 1330823

[84] Endoh M Zhu W Hasegawa J Watanabe H Kim DK Aida M. Human Spt6 Stimulates Transcription Elongation by RNA Polymerase II *In Vitro* . Mol Cell Biol (2004) 24(8):3324–36. doi: 10.1128/mcb.24.8.3324-3336.2004 PMC381665 15060154

[85] Le Bon A Tough DF. Links Between Innate and Adaptive Immunity *via* Type I Interferon. Curr Opin Immunol (2002) 14(4):432–6. doi: 10.1016/s0952-7915(02)00354-0 12088676

[86] Lin JP Fan YK Liu HM. The 14-3-3η Chaperone Protein Promotes Antiviral Innate Immunity *via* Facilitating MDA5 Oligomerization and Intracellular Redistribution. PloS Pathog (2019) 15(2):e1007582. doi: 10.1371/journal.ppat.1007582 30742689 PMC6386420

[87] Paludan SR Bowie AG Horan KA Fitzgerald KA. Recognition of Herpesviruses by the Innate Immune System. Nat Rev Immunol (2011) 11(2):143–54. doi: 10.1038/nri2937 PMC3686362 21267015

[88] Ma Z Ni G Damania B. Innate Sensing of DNA Virus Genomes. Annu Rev Virol (2018) 5(1):341–62. doi: 10.1146/annurev-virology-092917-043244 PMC6443256 30265633

[89] Kurt-Jones EA Orzalli MH Knipe DM. Innate Immune Mechanisms and Herpes Simplex Virus Infection and Disease. Adv Anatomy Embryol Cell Biol (2017) 223:49–75. doi: 10.1007/978-3-319-53168-7_3 PMC7254490 28528439

[90] Schoggins JW Rice CM. Interferon-Stimulated Genes and Their Antiviral Effector Functions. Curr Opin Virol (2011) 1(6):519–25. doi: 10.1016/j.coviro.2011.10.008 PMC3274382 22328912

[91] Pichlmair A Reis e Sousa C. Innate Recognition of Viruses. Immunity (2007) 27(3):370–83. doi: 10.1016/j.immuni.2007.08.012 17892846

[92] Desmet CJ Ishii KJ. Nucleic Acid Sensing at the Interface Between Innate and Adaptive Immunity in Vaccination. Nat Rev Immunol (2012) 12(7):479–91. doi: 10.1038/nri3247 22728526

[93] Suhara W Yoneyama M Kitabayashi I Fujita T. Direct Involvement of CREB-Binding Protein/P300 in Sequence-Specific DNA Binding of Virus-Activated Interferon Regulatory Factor-3 Holocomplex. J Biol Chem (2002) 277(25):22304–13. doi: 10.1074/jbc.M200192200 11940575

[94] Zhang Z Zheng Z Luo H Meng J Li H Li Q. Human Bocavirus NP1 Inhibits IFN-Beta Production by Blocking Association of IFN Regulatory Factor 3 With IFNB Promoter. J Immunol (Baltimore Md: 1950) (2012) 189(3):1144–53. doi: 10.4049/jimmunol.1200096 22745372

[95] Zhang M Liu Y Wang P Guan X He S Luo S. HSV-2 Immediate-Early Protein US1 Inhibits IFN-β Production by Suppressing Association of IRF-3 With IFN-β Promoter. J Immunol (Baltimore Md: 1950) (2015) 194(7):3102–15. doi: 10.4049/jimmunol.1401538 25712217

[96] Cloutier N Flamand L. Kaposi Sarcoma-Associated Herpesvirus Latency-Associated Nuclear Antigen Inhibits Interferon (IFN) Beta Expression by Competing With IFN Regulatory Factor-3 for Binding to IFNB Promoter. J Biol Chem (2010) 285(10):7208–21. doi: 10.1074/jbc.M109.018838 PMC2844170 20048166

[97] Melroe GT DeLuca NA Knipe DM. Herpes Simplex Virus 1 has Multiple Mechanisms for Blocking Virus-Induced Interferon Production. J Virol (2004) 78(16):8411–20. doi: 10.1128/JVI.78.16.8411-8420.2004 PMC479070 15280450

[98] Saira K Zhou Y Jones C. The Infected Cell Protein 0 Encoded by Bovine Herpesvirus 1 (Bicp0) Induces Degradation of Interferon Response Factor 3 and, Consequently, Inhibits Beta Interferon Promoter Activity. J Virol (2007) 81(7):3077–86. doi: 10.1128/JVI.02064-06 PMC1866033 17215277

[99] Orzalli MH DeLuca NA Knipe DM. Nuclear IFI16 Induction of IRF-3 Signaling During Herpesviral Infection and Degradation of IFI16 by the Viral ICP0 Protein. Proc Natl Acad Sci USA (2012) 109(44):E3008–17. doi: 10.1073/pnas.1211302109 PMC3497734 23027953

[100] Johnson KE Song B Knipe DM. Role for Herpes Simplex Virus 1 ICP27 in the Inhibition of Type I Interferon Signaling. Virology (2008) 374(2):487–94. doi: 10.1016/j.virol.2008.01.001 PMC2638114 18279905

[101] Zhang M Fu M Li M Hu H Gong S Hu Q. Herpes Simplex Virus Type 2 Inhibits Type I IFN Signaling Mediated by the Novel E3 Ubiquitin Protein Ligase Activity of Viral Protein Icp22. J Immunol (Baltimore Md: 1950) (2020) 205(5):1281–92. doi: 10.4049/jimmunol.2000418 32699158

[102] Liesegang TJ. Herpes Simplex Virus Epidemiology and Ocular Importance. Cornea (2001) 20(1):1–13. doi: 10.1097/00003226-200101000-00001 11188989

[103] Farooq AV Shukla D. Herpes Simplex Epithelial and Stromal Keratitis: An Epidemiologic Update. Surv Ophthalmol (2012) 57(5):448–62. doi: 10.1016/j.survophthal.2012.01.005 PMC3652623 22542912

[104] Chew T Taylor KE Mossman KL. Innate and Adaptive Immune Responses to Herpes Simplex Virus. Viruses (2009) 1(3):979–1002. doi: 10.3390/v1030979 21994578 PMC3185534

[105] Colonna M Pulendran B Iwasaki A. Dendritic Cells at the Host-Pathogen Interface. Nat Immunol (2006) 7(2):117–20. doi: 10.1038/ni0206-117 16424884

[106] Wingren AG Parra E Varga M Kalland T Sjogren HO Hedlund G. T Cell Activation Pathways: B7, LFA-3, and ICAM-1 Shape Unique T Cell Profiles. Crit Rev Immunol (2017) 37(2-6):463–81. doi: 10.1615/CritRevImmunol.v37.i2-6.130 29773030

[107] Sharpe AH Freeman GJ. The B7-CD28 Superfamily. Nat Rev Immunol (2002) 2(2):116–26. doi: 10.1038/nri727 11910893

[108] Matundan HH Jaggi U Wang S Ghiasi H. Loss of ICP22 in HSV-1 Elicits Immune Infiltration and Maintains Stromal Keratitis Despite Reduced Primary and Latent Virus Infectivity. Invest Ophthalmol Visual Sci (2019) 60(10):3398–406. doi: 10.1167/iovs.19-27701 PMC6685448 31387116

[109] Tormanen K Wang S Ghiasi H. CD80 Plays a Critical Role in Increased Inflammatory Responses in Herpes Simplex Virus 1-Infected Mouse Corneas. J Virol (2020) 94(2):e01511–19. doi: 10.1128/JVI.01511-19 PMC6955247 31619558

[110] Matundan H Ghiasi H. Herpes Simplex Virus 1 ICP22 Suppresses CD80 Expression by Murine Dendritic Cells. J Virol (2019) 93(3)e01803–18. doi: 10.1128/jvi.01803-18 PMC6340034 30404803

[111] Matundan HH Wang S Jaggi U Yu J Ghiasi H. Suppression of CD80 Expression by ICP22 Affects Herpes Simplex Virus Type 1 Replication and CD8(+)IFN-γ(+) Infiltrates in the Eyes of Infected Mice But Not Latency Reactivation. J Virol (2021) 95(19):e0103621. doi: 10.1128/jvi.01036-21 34287036 PMC8428405

[112] Livingston CM Ifrim MF Cowan AE Weller SK. Virus-Induced Chaperone-Enriched (VICE) Domains Function as Nuclear Protein Quality Control Centers During HSV-1 Infection. PloS Pathog (2009) 5(10):e1000619. doi: 10.1371/journal.ppat.1000619 19816571 PMC2752995

[113] Bastian TW Livingston CM Weller SK Rice SA. Herpes Simplex Virus Type 1 Immediate-Early Protein ICP22 Is Required for VICE Domain Formation During Productive Viral Infection. J Virol (2010) 84(5):2384–94. doi: 10.1128/jvi.01686-09 PMC2820935 20032172

[114] Adlakha M Livingston CM Bezsonova I Weller SK. The Herpes Simplex Virus 1 Immediate Early Protein ICP22 Is a Functional Mimic of a Cellular J Protein. J Virol (2020) 94(4):e01564–19. doi: 10.1128/jvi.01564-19 PMC6997750 31748398

[115] Kobayashi Y Kume A Li M Doyu M Hata M Ohtsuka K. Chaperones Hsp70 and Hsp40 Suppress Aggregate Formation and Apoptosis in Cultured Neuronal Cells Expressing Truncated Androgen Receptor Protein With Expanded Polyglutamine Tract. J Biol Chem (2000) 275(12):8772–8. doi: 10.1074/jbc.275.12.8772 10722721

[116] Zhang Y Yang Z Cao Y Zhang S Li H Huang Y. The Hsp40 Family Chaperone Protein DnaJB6 Enhances Schlafen1 Nuclear Localization Which is Critical for Promotion of Cell-Cycle Arrest in T-Cells. Biochem J (2008) 413(2):239–50. doi: 10.1042/bj20071510 18373498

[117] Meng E Shevde LA Samant RS. Emerging Roles and Underlying Molecular Mechanisms of DNAJB6 in Cancer. Oncotarget (2016) 7(33):53984–96. doi: 10.18632/oncotarget.9803 PMC5288237 27276715

[118] Majeski AE Dice JF. Mechanisms of Chaperone-Mediated Autophagy. Int J Biochem Cell Biol (2004) 36(12):2435–44. doi: 10.1016/j.biocel.2004.02.013 15325583

[119] Kon M Cuervo AM. Chaperone-Mediated Autophagy in Health and Disease. FEBS Lett (2010) 584(7):1399–404. doi: 10.1016/j.febslet.2009.12.025 PMC2843772 20026330

[120] Agarraberes FA Dice JF. A Molecular Chaperone Complex at the Lysosomal Membrane is Required for Protein Translocation. J Cell Sci (2001) 114(Pt 13):2491–9. doi: 10.1242/jcs.114.13.2491 11559757

[121] Zhou D Blum JS. Presentation of Cytosolic Antigens *via* MHC Class II Molecules. Immunol Res (2004) 30(3):279–90. doi: 10.1385/ir:30:3:279 15531770

[122] Deffit SN Blum JS. A Central Role for HSC70 in Regulating Antigen Trafficking and MHC Class II Presentation. Mol Immunol (2015) 68(2 Pt A):85–8. doi: 10.1016/j.molimm.2015.04.007 PMC4623969 25953005

[123] Attanasio R Olson R Johnson JC. Improvement in Plaquing Methods for the Enumeration of Anatid Herpesvirus (Duck Plague Virus). Intervirology (1980) 14(5-6):245–52. doi: 10.1159/000149193 7251330

[124] Nguyen ML Blaho JA. Apoptosis During Herpes Simplex Virus Infection. Adv Virus Res (2006) 69:67–97. doi: 10.1016/s0065-3527(06)69002-7 17222692

[125] Aubert M Krantz EM Jerome KR. Herpes Simplex Virus Genes Us3, Us5, and Us12 Differentially Regulate Cytotoxic T Lymphocyte-Induced Cytotoxicity. Viral Immunol (2006) 19(3):391–408. doi: 10.1089/vim.2006.19.391 16987059

[126] Aubert M Chen Z Lang R Dang CH Fowler C Sloan DD. The Antiapoptotic Herpes Simplex Virus Glycoprotein J Localizes to Multiple Cellular Organelles and Induces Reactive Oxygen Species Formation. J Virol (2008) 82(2):617–29. doi: 10.1128/JVI.01341-07 PMC2224592 17959661

[127] Jerome KR Chen Z Lang R Torres MR Hofmeister J Smith S. HSV and Glycoprotein J Inhibit Caspase Activation and Apoptosis Induced by Granzyme B or Fas. J Immunol (Baltimore Md: 1950) (2001) 167(7):3928–35. doi: 10.4049/jimmunol.167.7.3928 11564811

[128] Munger J Hagglund R Roizman B. Infected Cell Protein No. 22 is Subject to Proteolytic Cleavage by Caspases Activated by a Mutant That Induces Apoptosis. Virology (2003) 305(2):364–70. doi: 10.1006/viro.2002.1728 12573581

[129] Richard A Tulasne D. Caspase Cleavage of Viral Proteins, Another Way for Viruses to Make the Best of Apoptosis. Cell Death Dis (2012) 3(3):e277. doi: 10.1038/cddis.2012.18 22402601 PMC3317351

[130] You Y Cheng AC Wang MS Jia RY Sun KF Yang Q. The Suppression of Apoptosis by α-Herpesvirus. Cell Death Dis (2017) 8(4):e2749. doi: 10.1038/cddis.2017.139 28406478 PMC5477576

[131] Kruse JP Gu W. Modes of P53 Regulation. Cell (2009) 137(4):609–22. doi: 10.1016/j.cell.2009.04.050 PMC3737742 19450511

[132] Menendez D Shatz M Resnick MA. Interactions Between the Tumor Suppressor P53 and Immune Responses. Curr Opin Oncol (2013) 25(1):85–92. doi: 10.1097/CCO.0b013e32835b6386 23150340

[133] Hagglund R Munger J Poon AP Roizman B. U(S)3 Protein Kinase of Herpes Simplex Virus 1 Blocks Caspase 3 Activation Induced by the Products of U(S)1.5 and U(L)13 Genes and Modulates Expression of Transduced U(S)1.5 Open Reading Frame in a Cell Type-Specific Manner. J Virol (2002) 76(2):743–54. doi: 10.1128/jvi.76.2.743-754.2002 PMC136838 11752164

[134] Toubi E Shoenfeld Y. Toll-Like Receptors and Their Role in the Development of Autoimmune Diseases. Autoimmunity (2004) 37(3):183–8. doi: 10.1080/08916930410001704944 15497450

[135] Courtois G Gilmore TD. Mutations in the NF-kappaB Signaling Pathway: Implications for Human Disease. Oncogene (2006) 25(51):6831–43. doi: 10.1038/sj.onc.1209939 17072331

[136] Huxford T Huang DB Malek S Ghosh G. The Crystal Structure of the IkappaBalpha/NF-kappaB Complex Reveals Mechanisms of NF-kappaB Inactivation. Cell (1998) 95(6):759–70. doi: 10.1016/s0092-8674(00)81699-2 9865694

[137] Jacobs MD Harrison SC. Structure of an IkappaBalpha/NF-kappaB Complex. Cell (1998) 95(6):749–58. doi: 10.1016/s0092-8674(00)81698-0 9865693

[138] Ambagala APN Cohen JI. Varicella-Zoster Virus IE63, a Major Viral Latency Protein, Is Required To Inhibit the Alpha Interferon-Induced Antiviral Response▿. J Virol (2007) 81(15):7844–51. doi: 10.1128/JVI.00325-07 PMC1951283 17507475

[139] García MA Gil J Ventoso I Guerra S Domingo E Rivas C. Impact of Protein Kinase PKR in Cell Biology: From Antiviral to Antiproliferative Action. Microbiol Mol Biol Rev (2006) 70(4):1032–60. doi: 10.1128/mmbr.00027-06 PMC1698511 17158706

[140] Clemens MJ Elia A. The Double-Stranded RNA-Dependent Protein Kinase PKR: Structure and Function. J Interferon Cytokine Res: Off J Int Soc Interferon Cytokine Res (1997) 17(9):503–24. doi: 10.1089/jir.1997.17.503 9335428

[141] Rhoads RE. Regulation of Eukaryotic Protein Synthesis by Initiation Factors. J Biol Chem (1993) 268(5):3017–20. doi: 10.1016/S0021-9258(18)53649-8 8428975

[142] Schneider RJ Mohr I. Translation Initiation and Viral Tricks. Trends Biochem Sci (2003) 28(3):130–6. doi: 10.1016/S0968-0004(03)00029-X 12633992

[143] He B Gross M Roizman B. The Gamma(1)34.5 Protein of Herpes Simplex Virus 1 Complexes With Protein Phosphatase 1alpha to Dephosphorylate the Alpha Subunit of the Eukaryotic Translation Initiation Factor 2 and Preclude the Shutoff of Protein Synthesis by Double-Stranded RNA-Activated Protein Kinase. Proc Natl Acad Sci USA (1997) 94(3):843–8. doi: 10.1073/pnas.94.3.843 PMC19601 9023344

[144] Cassady KA Gross M. The Herpes Simplex Virus Type 1 U(S)11 Protein Interacts With Protein Kinase R in Infected Cells and Requires a 30-Amino-Acid Sequence Adjacent to a Kinase Substrate Domain. J Virol (2002) 76(5):2029–35. doi: 10.1128/jvi.76.5.2029-2035.2002 PMC135940 11836380

[145] Verweij MC Wellish M Whitmer T Malouli D Lapel M Jonjić S. Varicella Viruses Inhibit Interferon-Stimulated JAK-STAT Signaling Through Multiple Mechanisms. PloS Pathog (2015) 11(5):e1004901. doi: 10.1371/journal.ppat.1004901 25973608 PMC4431795

[146] Gray WL. Simian Varicella: A Model for Human Varicella-Zoster Virus Infections. Rev Med Virol (2004) 14(6):363–81. doi: 10.1002/rmv.437 15386593

[147] Kinchington PR Leger AJ Guedon JM Hendricks RL. Herpes Simplex Virus and Varicella Zoster Virus, the House Guests Who Never Leave. Herpesviridae (2012) 3(1):5. doi: 10.1186/2042-4280-3-5 22691604 PMC3541251

[148] James SF Mahalingam R Gilden D. Does Apoptosis Play a Role in Varicella Zoster Virus Latency and Reactivation? Viruses (2012) 4(9):1509–14. doi: 10.3390/v4091509 PMC3499816 23170169

[149] Hood C Cunningham AL Slobedman B Arvin AM Sommer MH Kinchington PR. Varicella-Zoster Virus ORF63 Inhibits Apoptosis of Primary Human Neurons. J Virol (2006) 80(2):1025–31. doi: 10.1128/jvi.80.2.1025-1031.2006 PMC1346839 16379003

[150] Zerboni L Sobel RA Ramachandran V Rajamani J Ruyechan W Abendroth A. Expression of Varicella-Zoster Virus Immediate-Early Regulatory Protein IE63 in Neurons of Latently Infected Human Sensory Ganglia. J Virol (2010) 84(7):3421–30. doi: 10.1128/jvi.02416-09 PMC2838126 20106930

[151] Cohrs RJ Gilden DH. Prevalence and Abundance of Latently Transcribed Varicella-Zoster Virus Genes in Human Ganglia. J Virol (2007) 81(6):2950–6. doi: 10.1128/jvi.02745-06 PMC1866015 17192313

